# Tunable Fluid-Type Metasurface for Wide-Angle and Multifrequency Water-Air Acoustic Transmission

**DOI:** 10.34133/2021/9757943

**Published:** 2021-09-30

**Authors:** Zhandong Huang, Shengdong Zhao, Yiyuan Zhang, Zheren Cai, Zheng Li, Junfeng Xiao, Meng Su, Qiuquan Guo, Chuanzeng Zhang, Yaozong Pan, Xiaobing Cai, Yanlin Song, Jun Yang

**Affiliations:** ^1^Department of Mechanical and Materials Engineering, The University of Western Ontario, London, Ontario, Canada N6A 5B9; ^2^School of Mathematics and Statistics, Qingdao University, Qingdao 266071, China; ^3^Institute of Mechanics for Multifunctional Materials and Structures, Qingdao University, Qingdao 266071, China; ^4^Key Laboratory of Green Printing, Institute of Chemistry, Chinese Academy of Sciences (ICCAS), Beijing Engineering Research Center of Nanomaterials for Green Printing Technology, Beijing National Laboratory for Molecular Sciences (BNLMS), Beijing 100190, China; ^5^Shenzhen Institute for Advanced Study, University of Electronic Science and Technology of China, Shenzhen 518000, China; ^6^Department of Civil Engineering, University of Siegen, D-57068 Siegen, Germany; ^7^Qingdao Branch of Institute of Acoustics, Chinese Academy of Sciences, Qingdao 266114, China

## Abstract

Efficient acoustic communication across the water-air interface remains a great challenge owing to the extreme acoustic impedance mismatch. Few present acoustic metamaterials can be constructed on the free air-water interface for enhancing the acoustic transmission because of the interface instability. Previous strategies overcoming this difficulty were limited in practical usage, as well as the wide-angle and multifrequency acoustic transmission. Here, we report a simple and practical way to obtain the wide-angle and multifrequency water-air acoustic transmission with a tunable fluid-type acoustic metasurface (FAM). The FAM has a transmission enhancement of acoustic energy over 200 times, with a thickness less than the wavelength in water by three orders of magnitude. The FAM can work at an almost arbitrary water-to-air incident angle, and the operating frequencies can be flexibly adjusted. Multifrequency transmissions can be obtained with multilayer FAMs. In experiments, the FAM is demonstrated to be stable enough for practical applications and has the transmission enhancement of over 20 dB for wide frequencies. The transmission enhancement of music signal across the water-air interface was performed to demonstrate the applications in acoustic communications. The FAM will benefit various applications in hydroacoustics and oceanography.

## 1. Introduction

Efficient communication across the water-air interface that covers over 70% of the Earth's surface has wide applications such as developing ocean networks [1], studying marine life [2–4], geological survey [5, 6], and remote sensing [7, 8]. Although both electromagnetic and acoustic waves can be used for communication in the air, acoustic waves are usually the only practical way to transmit information underwater because electromagnetic waves are rapidly attenuated [9]. Thereby, acoustic waves are an effective tool to connect the ocean, atmosphere, and land. However, when a sound impinges on the water-air interface, only 0.1% of the acoustic energy can be transmitted (namely, 30 dB loss) due to a large ratio of about 3600 in the acoustic impedances [10]. Acoustic metamaterials are promising to solve this challenge owe to the significant advantages in subwavelength manipulation of sound waves [11–13], whereas current acoustic metamaterials are usually used in a homogenous media such as air and water [14–16]. Their resonant elements such as tensioned membranes [15, 17, 18], elastic plates [19], Helmholtz resonators [16, 20], coiling-up space structure [21], and Mie resonators [22] are difficult to be constructed on the free water-air interface for acoustic transmission due to the interface instability, especially when metamaterials have a density larger than that of water. Although some efforts were made to achieve the water-air acoustic transmission, for example, utilization of evanescent plane waves [23, 24], membrane-type metasurface [10], and coupled resonant bubbles [25–27], there are no simple and practical methods yet to realize efficient water-air acoustic transmission, especially enabling the wide-angle and multifrequency transmission.

Here, we report a simple method to achieve the wide-angle and multifrequency water-air acoustic transmission by locking an air layer underwater using a hydrophobic solid substrate consisting of many hollow cells. The air in each cell and the upper water form a meta-atom of the fluid-type acoustic metasurface (FAM). The FAM has a thickness of less than a thousandth of the wavelength of sound in water, with the operating frequency range of 10 Hz~4000 Hz and energy transmission enhancement of over 200 times. We demonstrate that the FAM is robust and tunable, namely, a single FAM can work at different frequencies just by changing the immersion depth of the solid substrate. The mechanism is that the operating frequency is sensitive to the immersion depth while the energy transmission coefficient is not. The FAM can also apply to the water-to-air wide-angle incidence, which benefits from the small critical angle of total reflection at the water-air interface. Besides, the multifrequency transmission can be achieved by multilayer FAMs, and the supertransmission frequencies can be predicted analytically. For practical applications, the FAM exhibits good disturbance resistance and over four months' stability. Experiments and simulations reveal the FAM has a transmission enhancement of over 20 dB for wide frequencies and an incident angle of 0 ~ 80°. By exhibiting the transmission enhancement of the music signal, we demonstrated that FAM has practical applications in acoustic communications and sensing.

## 2. Results

### 2.1. Design of the FAM

**Figure 1:**
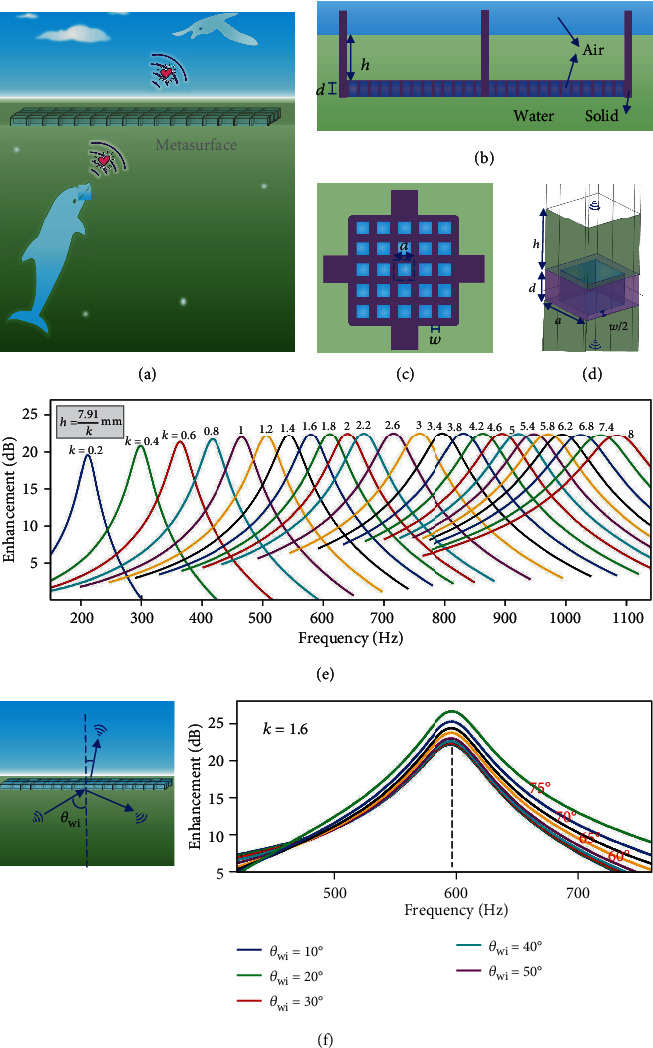
The design of the fluid-type metasurface for water-air acoustic transmission. (a) The FAM enables the communication between fish and birds. (b) and (c) The side and top view of the structure of metasurface. (d) The unit of the FAM for finite element method (FEM) calculations. (e) The transmission enhancement of the FAM vs. frequency is obtained with the FEM calculations. The parameters of *d*, *a*, and *w* are fixed as 5.11 mm, 3 mm, and 1.2 mm, respectively. The variable *h* is defined by the different *k*. (f) The FEM results for the water-to-air oblique incidence with an angle of *θ*_*wi*_. The *k* is fixed as 1.6, and other parameters are same as in (e). The thermal and viscous losses are considered in (e) and (f).

A poem said that the furthest distance in the world is that of fish and birds. Actually, the “remote distance” is not only from their entirely different habitats but also from the difficult acoustic communication across the air-water interface [28]. Here, we construct a FAM that enables an efficient sound transmission across the air-water interface (Figure 1(a)). Detailed design of the FAM is shown in Figures 1(b) and 1(c). An air layer with a thickness of *d* is trapped into the hydrophobic solid structure that contains arrays of hollow cells. The cuboid cells have the height of *d* and side length of *a*, and solid walls among cells have the thickness of *w*. Four square prisms were added at side edges to precisely control the immersion depth of *h* by balancing gravity and buoyancy force (Note S1). The air bubbles in the cells and the upper water serve as meta-atoms of the FAM [10]. The frequencies of enhanced transmission are determined by *h*, *d*, *a*, and *w* (Figure 1(d)). Once the solid structure is immersed into water, the air layer will be automatically located at a certain immersion depth, and the frequencies of enhanced transmission are decided. Besides, the operating frequency of the FAM is tunable [29]. By changing the immersion depth manually, the FAM can work at various frequencies (Figure 1(e)). It suggests that the FAM can enhance the sound transmission over 20 dB over a large range of frequencies. Moreover, the transmission enhancing effect still exists for the oblique incidence from 0 to 75° (Figure 1(f)).

### 2.2. Principle of the FAM

**Figure 2:**
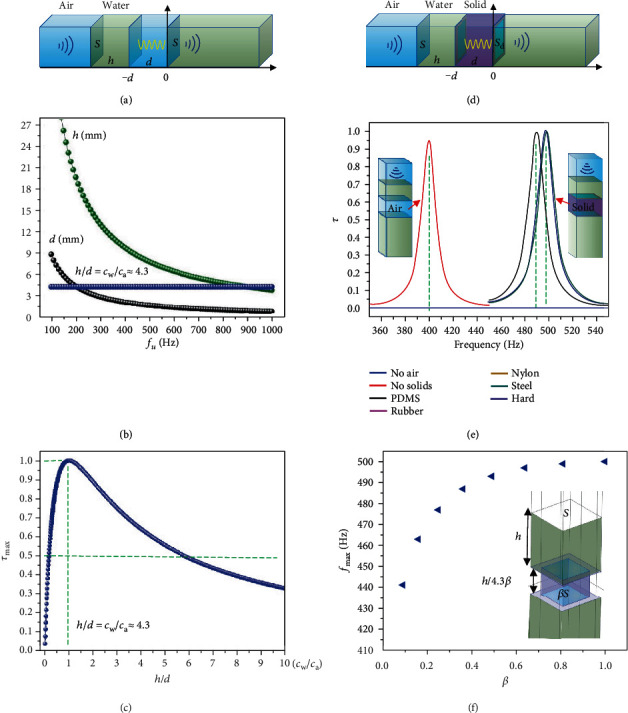
The principle of the FAM. (a) Schematic illustration of the ideal FAM without solid structure. (b) The frequency of unity transmission (*f*_*u*_) varying with *h* and *d* with the IMC calculation. (c) The maximum energy transmission coefficient (*τ*_max_) varying with *h*/*d* (Note S4). (d) Schematic illustration of the real FAM. The cross-sectional area changes from *S* to *S*_*d*_ due to the solid structure. (e) The effect of solid properties on the *f*_max_ shift. The parameters are *h* = 7.91 mm and *d* = 1.84 mm for the case without the solid, and *a* = 1.5 mm and *w* = 0.3 mm are added for the case with the solid. It suggests the effect of solid properties on *f*_max_ can be neglected if the solid is harder than the rubber. The notation “hard” represents the hard boundary condition. (f) The *f*_max_ varies with *β* by the FEM calculations. When *β* is small, the vibration of the water layer is confined by the solid structure, hence, *f*_max_ decreases as *β* decreases. This effect can be negligible when *β* > 0.64. The parameters are *h* = 7.91 mm, *a* = 3 mm, *d* = *h*/4.3*β*, and $w=a\left(1-\sqrt{\beta }\right)$, while *d* and *w* are variable with *β* for satisfying *h*/*βd* = *c*_*w*_/*c*_*a*_. The analytical model always predicts that *f*_max_ (about 500 Hz) regardless of *β* because it does not consider the solid constraint effect.

To explain the anomalous transmission above, we first consider the ideal case that a flat air layer is inserted into water (Figure 2(a)). For simplification, the thermoviscous loss is not considered at this stage until discussing acoustic experiments. When the sound from air impinges on the structure in Figure 2(a), the water layer will oscillate integrally because the thickness of *h* is much less than the wavelength, and the air is compressed or expanded with the water layer. Namely, the water serves as a mass and the air as a spring. Consequently, the propagation of sound in the water layer can be neglected. The assumption above is similar to that of Helmholtz resonators [16] and hybrid resonance of membrane-type acoustic metamaterials [10, 18]. The acoustic impedance of the mass-spring system (*Z*_*d*_) can be represented by the impedance at *x* = −*d* with the equation [10] (Note S2),

(1) $$\begin{array}{c} Z_{d}=\frac{Z_{\text{w}}}{1+\left(Z_{w}^{2}/Z_{a}^{2}-1\right)\sin^{2}\left(k_{a}d\right)}+i\left(\frac{k_{a}h\rho_{w}c_{a}}{S}-\frac{Z_{a}}{2}\frac{\left(Z_{w}^{2}/Z_{a}^{2}-1\right)\sin\left(2k_{a}d\right)}{1+\left(Z_{w}^{2}/Z_{a}^{2}-1\right)\sin^{2}\left(k_{a}d\right)}\right), \end{array}$$

where *Z*_*a*_ = *ρ*_*a*_*c*_*a*_/*S* and *Z*_*w*_ = *ρ*_*w*_*c*_*w*_/*S* are the acoustic impendence of air and water, respectively, *ρ*_*a*_, *c*_*a*_ and *ρ*_*w*_, *c*_*w*_ are the mass density and phase velocity of air and water, respectively. *k*_*a*_ is the wavenumber of sound traveling in air, and *S* is the cross-sectional area of the air and water layer. With the impedance matching condition (IMC) that *Z*_*d*_ = *Z*_*a*_, and the constraints that *Z*_*w*_ > >*Z*_*a*_ and *k*_*a*_*d* < <1, the frequency of unity transmission (*f*_*u*_) can be obtained as $f_{u}=c_{\text{a}}/2\pi \sqrt{\rho_{\text{a}}/hd\rho_{\text{w}}}$, with the precondition that *h*/*d* = *c*_*w*_/*c*_*a*_. The *f*_*u*_ also equals the natural resonant frequency of the water-air mass-spring system (Note S2). The relation of *h*, *d*, and *f*_*u*_ is plotted in Figure 2(b). For the general case, an analytical model (Note S3) was made to calculate the energy transmission coefficient (*τ*) for other frequencies,

(2) $$\begin{array}{c} \tau =\frac{4Z_{w}/Z_{a}}{{\left[\left(1+\left(Z_{w}/Z_{a}\right)\right)\cos k_{a}d-\left({\omega \rho }_{w}{hZ}_{w}/{SZ}_{a}^{2}\right)\sin k_{a}d\right]}^{2}+{\left[\left(1+\left(Z_{w}/Z_{a}\right)\right)\sin k_{a}d+\left({\omega \rho }_{w}h/{SZ}_{a}\right)\cos k_{a}d\right]}^{2}}, \end{array}$$

where *ω* is the angular frequency of the sound. The analytical model agrees well with the IMC and FEM calculations (Figure S1). By taking the first derivative of equation (2) with respect to frequency, we found that the frequency (*f*_max_) of the maximum energy transmission coefficient (*τ*_max_) exactly equals to the *f*_*u*_, and the corresponding *τ*_max_ is $\tau_{\max}=4c_{\text{w}}/c_{\text{a}}/{\left(c_{\text{w}}/c_{\text{w}}\sqrt{d/h}+\sqrt{h/d}\right)}^{2}$ (Note S4). The relation between *τ*_max_ and *h*/*d* is plotted in Figure 2(c), and *τ*_max_ reaches 1 when *h*/*d* = *c*_*w*_/*c*_*a*_. The results suggest, for the ideal water-air mass-spring system, the maximum transmission always occurs at its natural resonant frequency. The value of *hd* determines the *f*_max_ while *h*/*d* decides the *τ*_max_. Only when the IMC of *h*/*d* = *c*_*w*_/*c*_*a*_ is satisfied, the *f*_*u*_ exists and accordingly, *τ*_max_ = 1. The curves in Figures 2(b) and 2(c) also suggest the *f*_max_ is sensitive to *h* and *d* but *τ*_max_ is not. For example, *τ*_max_ remains over 50% for *h*/*d* varying from 0.3 to 25. Therefore, the FAM is robust and tunable. By varying the immersion depth *h*, the FAM can work at different frequencies with an insignificant influence on *τ*_max_ (Figure 1(e)).

### 2.3. Effect of the Solid Structure

The ideal water-air metasurface above is impractical because of the fluid instability from buoyancy of the air layer. Thus, we use a hydrophobic solid structure to trap the air layer (Figure 2(d)). The effect of the solid structure on the acoustic transmission should be firstly considered. Figure 2(e) suggests the shift of *f*_max_ mainly arises from the volume change of air in the cell because the cross-sectional area shrinks from *S* to *S*_*d*_, while the solid properties such as elastic modulus contribute little to the *f*_max_ shift. To modify the model, a parameter *β* = *S*_*d*_/*S* is defined, and the corresponding *Z*_*d*_ and *τ* are modified accordingly (Note S5). The maximum transmission frequency changes to $f_{\max}=c_{\text{a}}/2\pi \sqrt{\rho_{\text{a}}/hd\beta \rho_{\text{w}}}$, and the IMC changes to *h*/*βd* = *c*_*w*_/*c*_*a*_. Therefore, the *f*_max_ is determined by *hβd*, and *τ*_max_ is decided by *h*/*βd*. The predictions above agree well with the analytical model and FEM calculations (Figure S2). However, when *β* is small, the analytical solution and the IMC calculations in Note S5 will deviate from the FEM calculations because the former consider no solid constraint effect. Actually, the vibration of water is constrained by the solid due to the large change of the cross-sectional area. Figure 2(f) shows the deviation will be obvious when *β* < 0.5. Without regard to this effect, the analytical solution can be used to predict the effect of *h*, *d*, *a*, *w* on the *f*_max_ and the corresponding *τ*_max_ (Figure S3).

### 2.4. Multifrequency Acoustic Transmission with Multilayer FAMs

**Figure 3:**
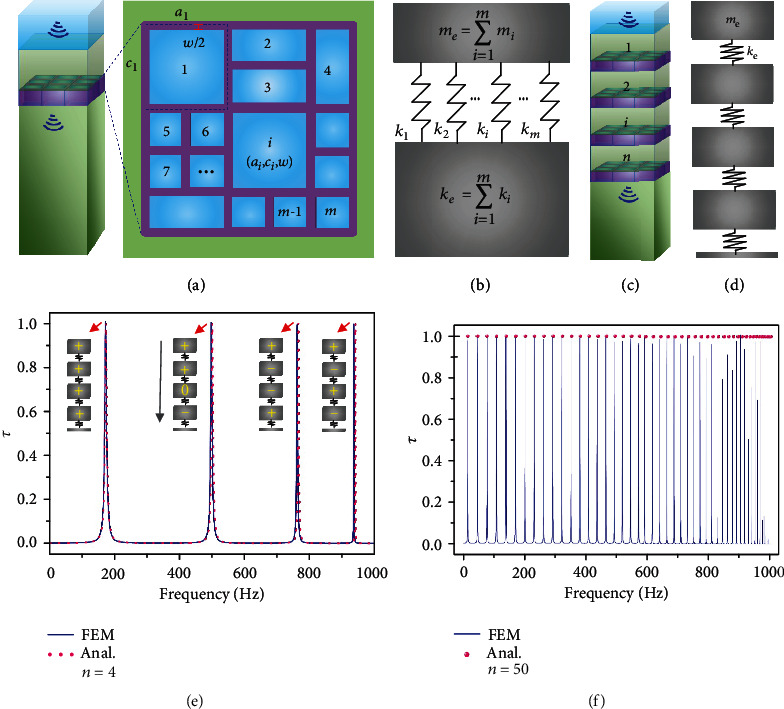
The parallel and series mass-spring model for predicting the *f*_max_. (a) Schematic illustration of the FAM with different bubble sizes. Every bubble in the cells is numbered. The *i*th bubble has the side lengths of *a*_*i*_ and *c*_*i*_, and the wall thickness is *w*, thus, the parameters for *i*th bubble can be marked as (*a*_*i*_, *c*_*i*_, *w*). (b) The structure in a can be treated as the parallel mass-spring system. The *m*_*e*_ and *k*_*e*_ are the effective mass and effective spring constant, respectively. (c) Schematic illustration of the multilayer structure of (a). The structure has *n* layer FAMs, and each water layer is numbered. (d) The structure in *c* can be treated as the series mass-spring system. (e) The energy transmission coefficient *τ* vs. frequency by the FEM and analytical solution in Note S3 for *n* = 4. The mass-spring models indicate the different modes of vibration at the corresponding resonant frequency. (f) The energy transmission coefficient *τ* vs. frequency for *n* = 50 obtained by the FEM calculations and the analytical solution from equations (4) and (5). The analytical solution predicts the frequencies of *f*_max_ very well.

It has been demonstrated an effective way to use the multiple resonators for the perfect multifrequency sound absorption [30]. Here, we demonstrate that it can also be used for multifrequency water-air acoustic transmission. As the natural resonant frequency of the water-air mass-spring system corresponds to the *f*_max_, therefore, *f*_max_ can be predicted by the vibration analysis. We first considered the hollow cells with different side lengths (marked with *a* and *c*) and the same wall thickness of *w* (Figure 3(a)). The equivalent mass-spring model is the parallel mass-spring system (Figure 3(b)). The natural frequency is the ratio of the sum of masses and the sum of spring constants [31, 32]. Combining mass and spring constant formula in Note S5 and *β*_*i*_ = (*a*_*i*_ − *w*)(*c*_*i*_ − *w*)/*a*_*i*_*c*_*i*_, the effective spring constant *k*_e_ = *ρ*_a_*c*_a_^2^∑_*i*=1_^*m*^*a*_*i*_^2^*c*_*i*_^2^/*d*(*a*_*i*_ − *w*)(*c*_*i*_ − *w*), and effective mass *m*_e_ = ∑_*i*=1_^*m*^*ha*_*i*_*c*_*i*_*ρ*_w_. The maximum transmission frequency (*f*_*e*max_) can be obtained as (Figure 3(b))

(3) $$\begin{array}{c} f_{e\max}=\frac{c_{a}}{2\pi }\sqrt{\frac{\rho_{a}}{\rho_{w}hd}}\sqrt{\overset{m}{\underset{i=1}{\sum }}\frac{a_{i}^{2}c_{i}^{2}/\left(a_{i}-w\right)\left(c_{i}-w\right)}{\sum_{i=1}^{i=m}a_{i}c_{i}}}, \end{array}$$

where *m* is the total number of cells. Equation (3) agrees well with the FEM calculations (Figure S4), thus, it can be used to predict the *f*_*e*max_ for cells with various different parameters. Next, the multilayer FAMs were considered for achieving the multifrequency supertransmission. The multilayer FAMs can be treated as the series mass-spring system (Figures 3(c) and 3(d)). We first considered the four layers with the analytical model in Note S3, and the energy transmission coefficient is obtained with iterative computations. The obtained analytical solution agrees well with FEM calculations (Figure 3(e)). The four-layer FAMs have four frequencies of unity transmission, and each of them corresponds to a mode of vibration (Figure S5). Assuming the direction of the air spring compression is positive, four modes of vibration can be marked as (+, +, +, +), (+, +, 0, −), (+, −, −, +), and (+, −, +, −). To predict the frequencies of unity transmission for the FAMs with an arbitrary finite number of *n* layers, the multidegree freedom mechanical vibration analysis [31, 32] is conducted in Note S6. Considering the simplest condition that all the water and air layers are, respectively, identical, and *ω*_0_ is the angular resonant frequency for one layer (*n* = 1), the angular resonant frequencies of unity transmission for the arbitrary *n*-layer FAMs can be expressed as

(4) $$\begin{array}{c} \omega =\omega_{0}\sqrt{2-\alpha -\alpha^{-1}}, \end{array}$$

where *α* satisfies the equation

(5) $$\begin{array}{c} \frac{\alpha^{2n+1}+1}{\alpha +1}=0. \end{array}$$

The *n*-layer structures have *n* angular frequencies of unity transmission, and all of them are between 0 and 2*ω*_0_, namely, 0 < *ω* ≤ 2*ω*_0_ (Note S6). Assuming that frequency of unity transmission for one layer is 500 Hz, by substituting *n* = 50 to equations (4) and (5), the 50 frequencies of unity transmission are obtained, which agree well with the FEM calculations (Figure 3(f)).

### 2.5. Preparation and Stability of the FAM

**Figure 4:**
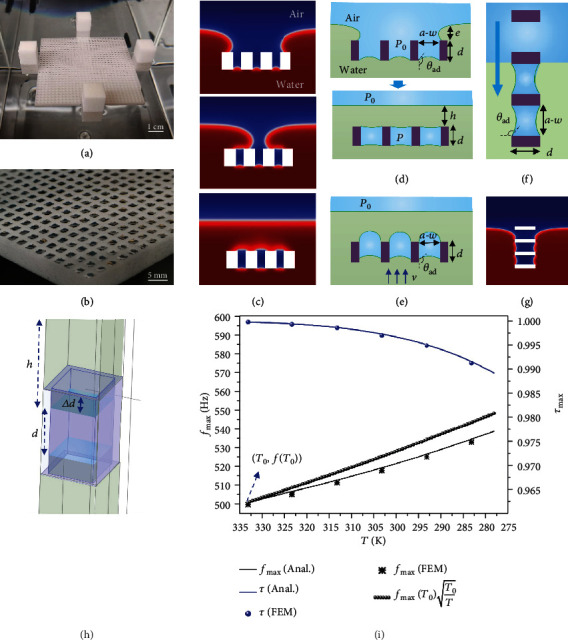
Preparation of the FAM and its stability. (a) The prepared FAM using the 3D printed solid structures. (b) The enlarged image of a shows trapped bubbles in the cells. (c) The FEM simulation for the bubble formation. See the Movie S2 for the full process. (d) The analysis of bubble formation process for the horizontal immersion method. The *P*_0_ is the atmospheric pressure, and *θ*_*ad*_ is the advancing angle on the solid surface. The *e* is the thickness of the water above the solid structure due to the balance of hydrophobic effect and gravity. *P* is the pressure in the bubble. (e) The critical state that the trapped bubbles will depart from the solid cell under a relative speed of *v* between water and the solid structure. The critical state for the bottom boundary is the contact angle larger than *θ*_*ad*_, and that for the upper boundary is the curvature radius equals (*a* − *w*)/2. (f) The vertical immersion method can be used for the large *d*, and the corresponding FEM simulation (g). See the Movie S6 for details. (h) When the temperature decreases, the *h* will increase and *d* will decrease due to the contraction of air, and *f*_max_ will shift slightly. (i) The temperature dependence of the FAM. The analytical solution and the FEM agree well.

Next, we prepared the FAM sample and analysed its stability for practical applications. Nylon material is selected for 3D printing because of its hydrophobicity, with the contact angle and advancing angle (*θ*_*ad*_) on the printed surface of 115 ± 5° and 135 ± 5°, respectively (Figure S6). The hydrophobicity enables it easy to trap air bubbles while being immersed in water (Movie S1). The solid structure is automatically immersed into a certain depth where the gravity balances the buoyancy force (Figure 4(a)). The air in each cell forms a closed bubble (Figure 4(b)), and the detailed formation process is shown by the FEM simulation (Figure 4(c) and Movie S2). For trapping the air layer successfully, the solid structure should obey two principles. First, the bubble size (*a* − *w*) should be smaller than the capillary length (about 2.7 mm) for the surface tension being dominated [33]. Besides, the maximum Laplace pressure at the bottom air-water interface should always exceed the liquid static pressure of *ρ*_*w*_(*e* + *d*)*g* during the formation process (Figure 4(d)), otherwise, water will penetrate into the cells [34, 35]. Therefore, the parameters *a*, *w*, and *d* should satisfy the inequation (Note S7)

(6) $$\begin{array}{c} \left(2k^{-1}\sin\frac{\theta_{ad}}{2}+d\right)\left(a-w\right)<-4k^{-2}\cos\theta_{ad}, \end{array}$$

where *k*^−1^ is the capillary length and equals $\sqrt{\sigma /\rho_{\text{w}}g}$, *σ* is the surface tension of water, and *g* is gravity acceleration. Once the bubbles are formed, they can be very stable. The immersion depth has a large influence during the bubble formation process because the process is isobaric. At first, the pressure in the cell is always *P*_0_, and if the immersion depth is large enough, the static pressure will exceed the maximum Laplace pressure (Figure 4(d)), then, water will penetrate into the cells. But once the bubbles are formed, the immersion will change to an isothermal process. The pressure *P* in the cell can increase by shrinking bubbles to resist the static pressure, which greatly weakens the influence of immersion depth. Actually, the closed bubbles even can sustain the vertical motion between the solid and water. The critical state that bubbles depart from the cells is shown as Figure 4(e). Using the dynamic pressure in Bernoulli equation [36], the allowed maximum speed *v*_max_ can be expressed as $v_{\max}=\sqrt{8\sigma \left(1-\cos\theta_{ad}\right)/\rho_{w}\left(a-w\right)-2gd}$(Note S8). The parameters in Figure 4(a) are *a* = 3 mm, *w* = 1.2 mm, *d* = 5.1 mm, and *θ*_*ad*_ = 135°. For *σ* = 72 mN/m, *g* = 9.8 N/kg, *ρ*_*w*_ = 998 kg/m^3^, the *v*_max_ is 0.67 m/s. The stability of the formed bubbles was demonstrated experimentally, and none of the shearing motion (Movie S3), vertical motion (Movie S4), and water wave disturbance (Movie S5) show obvious influence on the bubbles. When *d* is large, satisfying equation (6) will be difficult, and the vertical immersion method can be used (Figures 4(f) and 4(g)). The corresponding constraint equation changes to $a-w<\sqrt{-8k^{-1}\cos\theta_{ad}}$ (Note S7). The advantage of this method is shown in Movie S6.

### 2.6. Temperature Dependence

In practical applications, the FAM mainly faces challenges in three aspects, the liquid pressure, air dissolution, and temperature change. The FAM is usually close to the water-air interface. The liquid pressure is consequently very small compared with the atmospheric pressure, and air is almost saturated in the water. Therefore, the effect of pressure and dissolution can be neglected. It was found that bubbles prepared four months ago were still stable, which confirms this assumption. By considering the effect of temperature on the bubble volume, the upper water layer (Figure 4(h)), the density, and acoustic speed, the temperature dependence of the FAM has been studied (Note S9). It suggests when the temperature varies from 60°C to 5°C, the *f*_max_ will increase by about 10%, and the corresponding *τ*_max_ undergoes little changes (Figure 4(i)). For simplification, the dependence of the maximum transmission frequency of *f*_max_(*T*) on temperature can be approximately expressed as $f_{\max}\left(T\right)\approx f_{\max}\left(T_{0}\right)\sqrt{T_{0}/T}$, where *T* < *T*_0_. The ultrathin property of the FAM and its operating frequency range are also important for practical applications. The ultrathin property can be characterized by *λ*_*w*_/*d*, where *λ*_*w*_ is the wavelength at *f*_max_ in water, and $\lambda_{w}/d=2\pi c_{\text{w}}\sqrt{\rho_{\text{w}}h\beta /\rho_{\text{a}}d}/c_{\text{a}}$. Provided that *h*/*βd* = *c*_*w*_/*c*_*a*_ is satisfied, the *λ*_water_/*d* = 1611*β*. The wavelength is usually over 1000 times larger than the printed thickness *d*, and the value can be larger for other structures (Figure S7). Considering the wavelength of sound in air (*λ*_air_), it shows that *λ*_air_ is also over 200 times larger than *d*. Because *d* is usually at the millimeter scale, the multilayer FAMs are suitable for achieving multifrequency transmission. Assuming the height of the structure by 3D printing can be up to 10 cm, and the water layer less than 1 mm is impractical, the operating frequency range is estimated as 10 Hz~4000 Hz (Figure S8).

### 2.7. Acoustic Performance of the FAM

**Figure 5:**
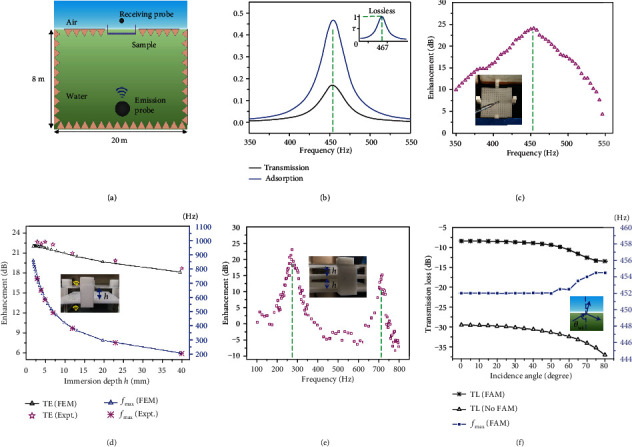
The acoustic performance of the FAM. (a) Schematic diagram of the experimental setup. (b) The FEM calculation for the energy transmission (absorption) coefficient vs. frequency. The parameters are the same as Figure 1(e) and *k* = 0.9. It suggests about 17% and 36% of the sound energy are transmitted and reflected, respectively, and 47% are dissipated. The inset shows the FEM result without thermal and viscous losses. (c) The experiments of the transmission enhancement with the FAM with the setup in (a). The *f*_max_ agrees well with that in (b). The inset is the FAM and the receiving probe. (d) The FEM calculations and experimental results for different immersion depths, with the same parameters as Figure 1(e). The “TE” is the abbreviation of transmission enhancement. The inset shows the variable *h*. (e) The experimental transmission enhancement vs. frequencies for two layers of FAM. The inset shows the two-layer FAMs. (f) FEM results for water-to-air oblique incidence with and without the FAM. It shows that the transmission enhancement always works for the oblique incidence, and *f*_max_ only slightly increases (blue line) by about 2 Hz. The “TL” is the abbreviation of transmission loss.

Next, we demonstrate the acoustic performance of the FAM. The setup is shown in Figure 5(a). The water pool has a size of 20 m × 12 m × 8 m, surrounding by the absorbing wedges (Figure S9). However, the setup is still not effective enough for absorbing the sound at near 450 Hz for preventing reflections. Thus, the precise energy transmission coefficient vs. frequency is hard to be obtained in experiments. Here, the setup is mainly used to measure the *f*_max_, and the transmission with and without the FAM is to qualitatively exhibit the transmission enhancement. Figure 5(b) is the FEM calculation of the transmission and absorption coefficient vs. frequency. It shows *τ*_max_ decreases from 100% to about 17%, and *f*_max_ decreases from 467 Hz to 452 Hz due to the thermoviscous loss [10]. There are about 36%, and 47% of the sound energy is dissipated and reflection because the thermoviscous loss has varied the IMC (Note S10). By varying the immersion depth, the energy transmission can be about 20%, which corresponds to the transmission enhancement of 22.5 dB (Figure 1(e)). Figure 5(c) shows the transmission enhancement with the FAM, and the *f*_max_ agrees well with the FEM calculation in Figure 5(b). Actually, the *f*_max_ still agrees with the FEM even a small water sink is used (Movie S7), and the qualitatively transmission comparison with and without the FAM is also shown (Movie S8). By varying the immersion depth in Figure 5(a), the *f*_max_ can be varied from 200 Hz to 800 Hz with the transmission enhancement of over 20 dB (Figure 5(d)). The noise source in a small sink was also used to confirm this performance qualitatively (Movie S9). Besides, the multifrequency transmission is also demonstrated in Figure 5(e). The parameters for each layer are the same with Figure 5(c). There are transmission enhancement of 23 dB and 15 dB at 273 Hz and 717 Hz, respectively. Equations (4) and (5) provide that the peaks are at 288 Hz and 755 Hz, and the difference between theory and experiments might arise from the dissipation.

For the oblique incidence, the FAM obeys the traditional Snell's law because the abrupt phase discontinuity along the interface does not change [13, 37]. Similar to the normal incidence, the IMC for the oblique incidence case is calculated (Note S11). The maximum transmission frequency for the oblique incidence (*f*_*s*max_) and the normal incidence (*f*_max_) obey the relation

(7) $$\begin{array}{c} f_{s\max}=\frac{f_{\max}}{\cos\theta_{ai}}=\frac{f_{\max}}{\sqrt{1-c_{a}^{2}\sin^{2}\left(\theta_{wi}/c_{w}^{2}\right)}}, \end{array}$$

where *θ*_*wi*_ and *θ*_*ai*_ are the incident and refraction angle form water to air, respectively. The *θ*_*ai*_ is also the incident angle from air to water according to Snell's law. The IMC changes to

(8) $$\begin{array}{c} \frac{h}{\beta d}=\frac{c_{w}\cos\theta_{ai}}{c_{a}\cos\theta_{wi}}. \end{array}$$

The critical angle of total reflection from air to water is very small, and *θ*_*ai*_ only changes from 0 to 13.4° when *θ*_*wi*_ varies from 0 to 90°. Therefore, the *f*_*s*max_ in equation (7) is nearly invariable with *θ*_*wi*_. However, *τ* decreases with the increase of *θ*_*wi*_, because the IMC for the normal incidence (*h*/*βd* = *c*_*w*_/*c*_*a*_) does not satisfy equation (8), especially when *θ*_*wi*_ is large. The transmission coefficient calculation for the oblique incidence (Note S12) agrees well with the conclusions above and FEM calculations (Figure S10). After considering the thermoviscous loss, the results still agree with the predictions above. Comparing to the case without FAM, the transmission enhancement for the oblique incidence is still more than 22 dB (Figure 5(f)) and even increase with the incident angle (Figure 1(f)). This is because the transmission for the bare water-air interface decreases more with the increasing of the incident angle than that of the case with FAM (Figure 5(f)).

### 2.8. The Application in Enhancing Acoustic Information Transmission

**Figure 6:**
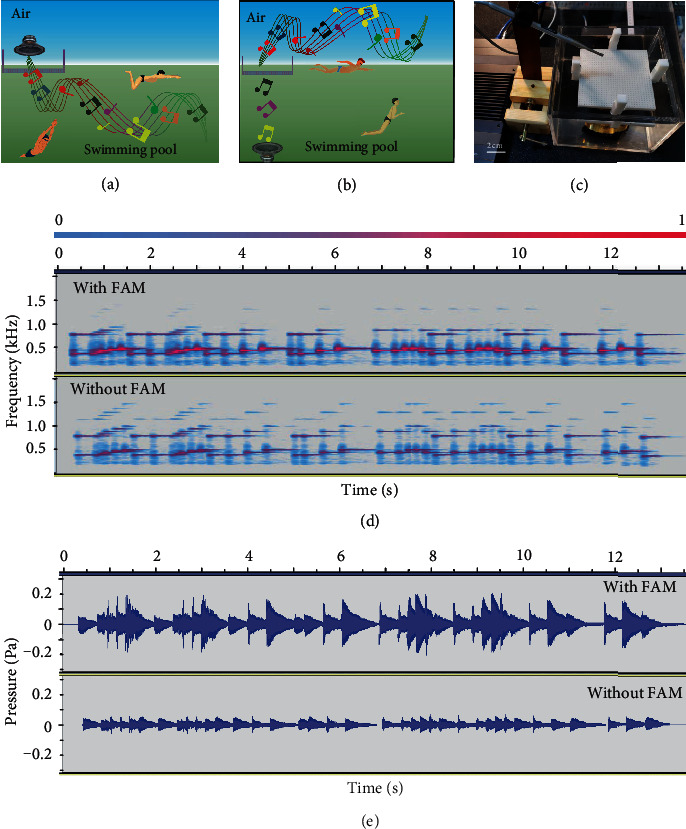
The application in enhancing the acoustic signal across the water-air interface. (a) The schematic diagram that an in-air loudspeaker might replace the expensive underwater speaker with the FAM. (b) The underwater speaker might work with a low power to emit the music from water to air with the FAM. (c) The experiment setup to demonstrate the FAM can enhance the music signal transmission across the water-air interface. (d) The received music signal from water to air with and without the FAM. The color from blue to pink exhibits the increase of the amplitude of acoustic pressure. It suggests that the fundamental frequencies of the music signal are enhanced with the FAM. (e) The acoustic pressure of the received music signal with and without the FAM.

The discussion above shows that the FAM can enhance the transmission of the acoustic energy. Next, we use the music signal as an example to demonstrate the acoustic information can also be enhanced. The practical applications include replacing the expensive underwater speaker in the swimming pool with a low-cost airborne speaker [38] (Figure 6(a)) and emitting the music from water to air with an underwater speaker of a low power (Figure 6(b)). To demonstrate that, we made a music signal with the fundamental frequencies from 350 Hz to 500 Hz (Figure S11), which is near the operating frequencies (*f*_max_ = 452 Hz) of the FAM (Figure 5(c)). The music signal was emitted in the water sink and received in air with or without the FAM (Figure 6(c)). The comparison with and without the FAM is shown in Movie S10. It suggests that the FAM can obviously enhance the transmission of the music signal. By analysing the received music signal, we can find that the fundamental frequency part of the music is enhanced with the FAM (Figure 6(d)). The comparison of the received acoustic pressure is shown in Figure 6(e), conforming that the music signal was enhanced by the FAM. The results above suggest that the FAM has the promising applications for the acoustic communications between ocean and atmosphere.

## 3. Discussion

There is a transmission loss of about 30 dB when the sound wave propagates across the water-air interface [28]. Compared with the absorption coefficient of seawater of about 0.025 dB/km at 500 Hz [39], the transmission loss of water-air interface nearly equals the loss from a transmission distance of 1200 km. Therefore, the water-air is a great barrier for the sound wave propagation. Here, the FAM was provided to break this barrier with a transmission enhancement over 20 dB, which means that it reduces the transmission loss from a transmission distance of 800 km. The FAM has many irreplaceable advantages over the traditional acoustic metasurface [15, 18, 40]. First, compared with the membrane-type metasurface that is based on the hybrid resonance of the membrane's two eigenmodes [10, 18], the resonance in the FAM is very simple, and the operating frequency can be well predicted. Second, by changing the immersion depth, the resonant frequency of the FAM can be flexibly adjusted, thus, a single FAM could operate at various frequencies, and the operating frequency can be tunable. Third, the FAM can achieve multifrequency transmission with multilayer bubbles, which is hard to achieve with current metasurface [10]. Last, the fabrication of FAM is simple and low-cost. It has no strict requirements for the 3D printing technology, and many materials can be used as long as the surface can be coated with a hydrophobic substance such as the fluorosilane [41].

In this work, we propose an efficient FAM for wide-angle and multifrequency water-air sound transmission. The FAM opens an acoustic window at the water-air interface for acoustic transmission, which might enable various applications that are infeasible so far. For example, it allows to characterize the underwater sound with airborne sound sensoring systems [10], so that the special underwater acoustic devices could become needless [42]. By using FAMs, airborne sound systems can simultaneously detect the sound both from water and air. Moreover, the measurement will have a higher signal-to-noise ratio due to the much lower ambient noise level in the atmosphere than that in the ocean [43]. Similarly, the FAM can also aid the underwater acoustic sensors to detect aircrafts [44]. Furthermore, the FAM will enhance the communication across the water-air interface, such as remote operation of underwater robots [45], information exchange between the submarine and aircraft [46], and remote sensing of sound in the ocean [7, 47]. Finally, the FAM allows the effective energy transmission from water to air; thus, the underwater acoustic energy can be harvested and transformed by the airborne piezoelectric transduction devices [48, 49]. The negative effect from static pressure and corrosivity of seawater can be avoidable [50, 51]. Thus, the FAM provides a promising platform for enhancing acoustic communications and sensing across the water-air interface and benefits various applications in marine biology and geology, remote sensing, energy conversion, communication engineering, etc.

## 4. Materials and Methods

### 4.1. Acoustic Experiments

The acoustic experiment facilities include the water pool, 3D printing nylon solid structure, acoustic source, B&K acoustic testing system, and the auxiliary clamping equipment. The solid structure was prepared with commercial 3D printing technology, with a density of about 1020 kg/m^3^ and the contact angle on the surface of about 115°. Actually, almost any material can be used as long as the surface can be coated with a hydrophobic substance. When being immersed in water, the hydrophobic surface enabled the nylon structure to trap bubbles in the hollow cells, and the density slightly higher than water made it possible to be automatically located at a certain depth by controlling the gravity and buoyancy force. For the acoustic experiments in Figures 5(c)–5(e), the water pool has a size of 20 m × 12 m × 8 m, surrounding by the absorbing wedges. The acoustic source and the power amplifier (NYk5887-L16) were shown in Figure S8. The transmission was measured at every two frequency points. The transmission enhancement was obtained by the difference of the acoustic transmission with and without the FAM. In Figure 5(d), the solid structure was controlled at different immersion depths, and the corresponding acoustic transmission enhancement and the *f*_max_ were measured. The transmission enhancement was defined by the difference of the acoustic transmission (dB) with and without the FAM. For all the acoustic measurements, the signals were averaged with 100 acquisitions. For the measurement of *f*_max_ and qualitative comparison with and without the FAM (Movie S7-10), the small water sink (13 cm × 13 cm × 13 cm) was used for flexibly manipulating the solid structure. The waterproof loudspeaker as an acoustic source was put into water. To prevent the vibration energy radiating outward through the base, the water sink and the acoustic source water were placed on acoustical sponges, respectively. The acoustic source signals including single frequency, scanning frequency, and broadband noise signals were generated by the signal editor, and the signals were transmitted to the underwater acoustic source via Bluetooth. The B&K acoustic testing system consists of a signal acquisition instrument (type 3160-A-042), a 1/8-inch sound microphone (type 4138-A-015), an acoustic signal analysis software, and the corresponding connecting wiring. In experimental tests, the microphone was placed above the water to pick up the transmitted acoustic signals across the FAM or the bare water-air interface. The signal acquisition instrument can collect the time-domain signal from the microphone and carry out the real-time Fourier transform and finally obtain the real-time changing transmission spectrum. In the acoustic experiments in Movie S7, the scanning frequency from 200 Hz to 700 Hz was used. In Movie S8, the transmission performance test with and without the FAM was conducted with the single frequency single about 450 Hz. In Movie S9, the noise source was used, and the immersion depth was adjusted manually to realize the operation at different frequencies. In Movie S10, the special music signal was used.

### 4.2. Acoustic Calculations

The numerical acoustic calculations are performed by COMSOL Multiphysics 5.4. For calculations of the ideal FAM without solid (Figure S1), the acoustic pressure module was used with the periodic boundaries and plane wave radiation conditions. The energy transmission coefficient was calculated by the ratio of *p*^2^/2*Z* of the incident and transmitted waves, where *p* is amplitude of the acoustic pressure and *Z* is the characteristic specific acoustic impedance of the corresponding media. For the real FAM with solid, the acoustic-solid interaction was additionally taken into account (Figure 2(e)). The solid part in other models (such as in Figure 2(f) and Figure S2) was replaced by the hard boundary conditions in the acoustic pressure module because the solid part had little influence on the acoustic transmission. The 2D model was used in Figure 3(f) for reducing the computational effort. For considering the thermal and viscous losses in Figures 1(e) and 1(f) and Figures 5(b), 5(d), and 5(e), the Acoustic-Thermoviscous Acoustic Interaction Module was used. The boundary layers were resolved by using the boundary layer mesh, and the solid wall was assumed to be isothermal and nonslip. For the water-to-air oblique incidence in Figure 1(f), Figure 5(f), and Figure S10, the background acoustic field was used. The incident angle was defined manually, and the corresponding Floquet periodicity boundary condition was set up. The energy transmission coefficient (*τ*_*s*_) for the oblique incidence was calculated by *τ*_*s*=_ *τ*cos*θ*_*ai*_/cos*θ*_*wi*_, where *τ* is the expression of energy transmission coefficient for the normal incidence above, and *θ*_*wi*_ (*θ*_*ai*_) is the incident (refraction) angle. The transmission loss in Figure 5(f) was defined as 10log_10_(*τ*_*s*_), where *τ*_*s*_ is the energy transmission coefficient above. The transmission enhancement is determined by the difference in the transmission losses with and without the FAM (namely, the bare water-air interface). The Matlab (Mathworks, Natick, MA) was used for plotting the diagrams in Figure S3, evaluating the analytical solution with iterative computations for Figure 3(e) and solving the polynomial equation for Figure 3(f).

### 4.3. FEM Simulations

The finite element simulations of the formation process of bubbles were carried out with the COMSOL Multiphysics 5.4. The Two-Phase Flow, Phase Field Module was used. The moving mesh was set at a velocity of 0.5 mm/s. The advancing angle of the solid surface was 135°. The 2D model was used for simplifications. The difference between the 2D and 3D models is that the Laplace pressure for the 2D model is *σ*/*R*, and that for the 3D model is *σ*/2*R*, where *σ* is the surface tension of water, and *R* is the curvature radius of the bottom water-air interface. It suggests that the 3D case allows a larger *a* − *w* than that of the 2D case because of its larger critical pressure that breaks the Cassie state.

## Data Availability

All data required to support the conclusions are presented in the main text and the supplementary materials.

## Supplementary Materials

Supplementary Materials Note S1: designing the gravity and buoyancy force to precisely control the immersion depth. Note S2: the ideal impedance matching FAM without solid structure. Note S3: the analytical model for calculating the transmission coefficients. Note S4: the analytical model agrees very well with calculations from impedance matching conditions. Note S5: the effect of the solid structure. Note S6: the multilayer FAMs: series mass-spring model. Note S7: the methods for trapping the air bubbles in water. Note S8: the stability analysis for the prepared bubbles. Note S9: the temperature dependence of the acoustic metasurface. Note S10: the effect of the thermoviscous loss on the energy transmission coefficient. Note S11: the impedance-matched FAM for the oblique incidence. Note S12: calculating the transmission coefficients for the oblique incidence. Figure S1: the analytical solution in Note S3 agrees well with the FEM and the IMC calculations. Figure S2: the analytical solution in Note S5 agrees well with the FEM and the impedance matching condition calculation after considering the effect of solid structure. Figure S3: the effects of *h*, *d*, *a*, and *w* on the maximum transmission frequency and the corresponding energy transmission coefficient. Figure S4: Bubbles with different parameters can be treated as the parallel mass-spring model. Figure S5: the modes of vibration at different frequencies of unity transmission for *n* = 4. Figure S6: the contact angle and the advancing angle of the printed nylon structure. Figure S7: the ultrathin property of the FAM. Figure S8: the operating frequency range for the FAM. Figure S9: the acoustic experiments for demonstrating the acoustic performance of the FAM. Figure S10: the analytical solution, IMC calculation, and FEM calculation for water-to-air oblique incident agree well. Figure S11: the melody of the music signal for the FAM. Movie S1: the preparation of the fluid-type acoustic metasurface. Movie S2: the FEM simulation of the formation of bubbles. Movie S3-5: the demonstration of the stability of the trapped bubbles with the shearing motion, vertical motion, and disturbance from water wave. Movie S6: the vertical immersion method could be used for the large d. Movie S7: the performance of the acoustic metasurface with the scanning frequency. Movie S8: the comparison between the case with and without the fluid-type acoustic metasurface (Movie with sound). Movie S9: the FAM can work at different frequencies by changing the immersion depth. Movie S10: the FAM can enhance the transmission of music signal across the water-air interface (Movie with sound).
