# Supplementary material for: Tunable Fluid-Type Metasurface for Wide-Angle and Multifrequency Water-Air Acoustic Transmission
Source: Research (Wash D C). 2021 Sep 30;2021:9757943. doi: 10.34133/2021/9757943 (PMC8501414; doi:10.34133/2021/9757943)
Supplement: Supplementary Materials — Note S1: designing the gravity and buoyancy force to precisely control the immersion depth. Note S2: the ideal impedance matching FAM without solid structure. Note S3: the analytical model for calculating the transmission coefficients. Note S4: the analytical model agrees very well with calculations from impedance matching conditions. Note S5: the effect of the solid structure. Note S6: the multilayer FAMs: series mass-spring model. Note S7: the methods for trapping the air bubbles in water. Note S8: the stability analysis for the prepared bubbles. Note S9: the temperature dependence of the acoustic metasurface. Note S10: the effect of the thermoviscous loss on the energy transmission coefficient. Note S11: the impedance-matched FAM for the oblique incidence. Note S12: calculating the transmission coefficients for the oblique incidence. Figure S1: the analytical solution in Note S3 agrees well with the FEM and the IMC calculations. Figure S2: the analytical solution in Note S5 agrees well with the FEM and the impedance matching condition calculation after considering the effect of solid structure. Figure S3: the effects of h, d, a, and w on the maximum transmission frequency and the corresponding energy transmission coefficient. Figure S4: Bubbles with different parameters can be treated as the parallel mass-spring model. Figure S5: the modes of vibration at different frequencies of unity transmission for n = 4. Figure S6: the contact angle and the advancing angle of the printed nylon structure. Figure S7: the ultrathin property of the FAM. Figure S8: the operating frequency range for the FAM. Figure S9: the acoustic experiments for demonstrating the acoustic performance of the FAM. Figure S10: the analytical solution, IMC calculation, and FEM calculation for water-to-air oblique incident agree well. Figure S11: the melody of the music signal for the FAM. Movie S1: the preparation of the fluid-type acoustic metasurface. Movie S2: the FEM simulation of the formation [file 9757943.f1.zip › Supporting Materials.docx]

**Supporting Materials for**

**Tunable fluid-type metasurface for wide-angle and multifrequency water-air acoustic transmission**

Zhandong Huang^1,8^, Shengdong Zhao^2,3,8^, Yiyuan Zhang^1^, Zheren Cai^4^, Zheng Li^4^, Junfeng Xiao^1^, Meng Su^4^, Qiuquan Guo^5^, Chuanzeng Zhang^6^, Yaozong Pan^7^, Xiaobing Cai^1^*, Yanlin Song^4^*, Jun Yang^1,5^*

^1^Department of Mechanical and Materials Engineering, The University of Western Ontario, London, Ontario N6A 5B9, Canada

^2^School of Mathematics and Statistics, Qingdao University, Qingdao 266071, China;

^3^Institute of Mechanics for Multifunctional Materials and Structures, Qingdao University, Qingdao 266071, China

^4^Key Laboratory of Green Printing, Institute of Chemistry, Chinese Academy of Sciences (ICCAS), Beijing Engineering Research Center of Nanomaterials for Green Printing Technology, Beijing National Laboratory for Molecular Sciences (BNLMS), Beijing, 100190, P. R. China.

^5^Shenzhen Institute for Advanced Study, University of Electronic Science and Technology of China, Shenzhen, 518000, P. R. China.

^6^Department of Civil Engineering, University of Siegen, D-57068 Siegen, Germany.

^7^Qingdao Branch of Institute of Acoustics, Chinese Academy of Sciences, Qingdao, 266114, P. R. China.

^8^These authors contributed equally: Z. Huang and S. Zhao.

^*^E-mail: [xcai32@uwo.ca](mailto:xcai32@uwo.ca), [ylsong@iccas.ac.cn](mailto:ylsong@iccas.ac.cn), [jyang@eng.uwo.ca](mailto:jyang@eng.uwo.ca)

**Supplementary Materials include:**

Note S1-12

Figure S1-11

Captions for Movie S1-10

Reference

**Note S1**

**Designing the gravity and buoyancy force to precisely control the immersion depth.**


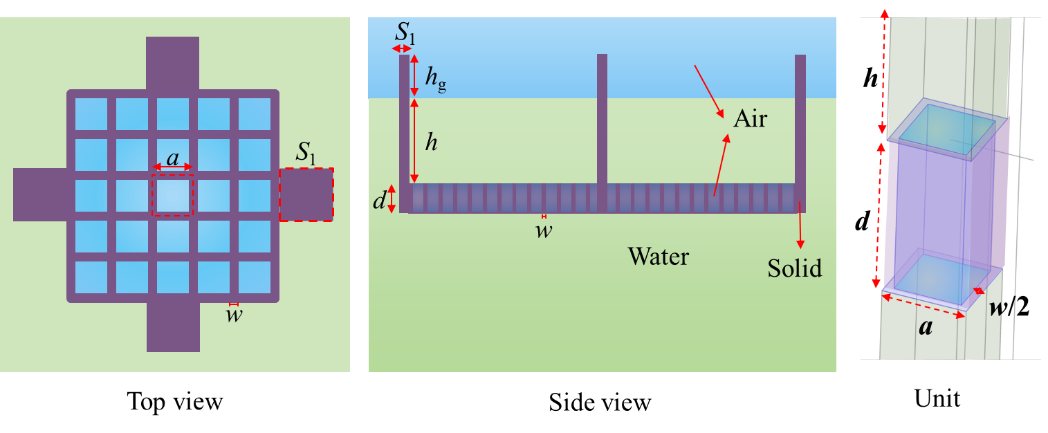


**Scheme S1.** The top view, side view and unit of the FAM

The parameters of *h*, *d*, *a*, *w* should be precisely controlled because they determine the maximum transmission frequency and the energy transmission coefficient. The *d*, *a*, *w* can be easily controlled because *a* is the side length, and *w, d* are the thickness and height of the wall among cells. The *h* is the thickness of the water layer and also the immersion depth of the solid structure. It can be precisely controlled by designing the buoyancy and gravity. As shown in scheme S1, the four regular square prisms are added to the edges of the solid structure. Part of the prism will be above the water, and the height is *h*_g_. The prism has a base area of *S*_1_ and height of *h*_g_ + *h* + *d*. The density of air, water, and solid are assumed to be *ρ*_a_, *ρ*_w_, *ρ*_s_, respectively, and there are *N*_c_ cells in total, the gravity *G* of the structure and the trapped air is

$G=[\rho_{\text{s}}d(2aw-w^{2})+\rho_{\text{a}}(a-w)^{2}d]N_{\text{c}}g+4\rho_{\text{s}}S_{1}(h+h_{g}\text{+}d)g$ (S1)

where *g* is the acceleration of gravity. The buoyancy force (*F*_b_) is

$F_{\text{b}}=\rho_{\text{w}}da^{2}N_{\text{c}}g+4\rho_{\text{w}}S_{1}(h+d)g$ (S2)

The gravity equals the buoyancy when achieving equilibrium, the following equation will be got

$h_{g}+h+d=\frac{\rho_{\text{w}}}{\rho_{\text{s}}}(\frac{da^{2}N_{\text{c}}}{4S_{1}}+h+d)-[2aw-w^{2}+\frac{\rho_{\text{a}}}{\rho_{\text{s}}}(a-w)^{2}]\frac{N_{\text{c}}d}{4S_{1}}$ (S3)

Substituting parameters that *ρ*_w_ = 998 Kg/m^3^, *ρ*_a_ = 1.21 Kg/m^3^, *ρ*_s_= 1020 Kg/m^3^_,_ *a* = 3 mm, *w* = 1.2 mm, *h* = 8.8 mm, *d* = 5.11 mm, *N*_c_ = 900, *S*_1_ = 144 mm^2^. The *h*_g_ is 24 mm. Namely, the 4 regular square prisms with a base area of 144 mm^2^ and a height of 37 mm should be added into cell structures that have an area of 90 mm×90 mm. The prisms will be automatically immersed into the depth of 8.8 mm in water, leaving the height of 24 mm above water. These parameters are used for acoustic experiments in Fig. 5.

**Note S2**

**The ideal impedance matching FAM without solid structure**

1. **The acoustic impedance at the surface of the FAM.**


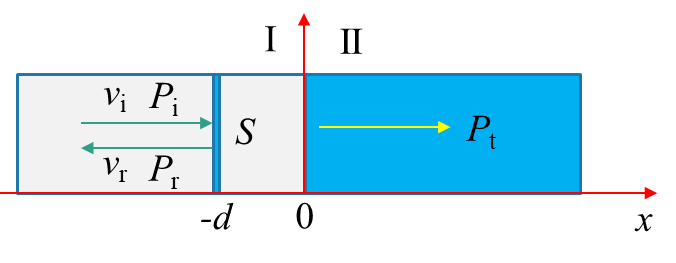


**Scheme S2.** The schematic diagram of the idea FAM. Phase I and II are air and water, respectively. The *P*_i_*, v*_i_*, and P*_r_*, v*_r_ are the acoustic pressure, particle velocity of the incident and reflected acoustic wave. The *S* is the cross-sectional area.

An air layer with the thick of *d* was inserted into the water at the position of *x* = 0. When a harmonic oscillation from air acts on the air-water interface, the water layer will oscillate integrally due to the thickness of the water layer is much less than the wavelength, and the air is compressed or expanded with the water layer. Namely, the water serves as a mass and the air as a spring. Consequently, the propagation of sound in the upper water layer can be neglected. The assumption above is similar with the Helmholtz resonators and hybrid resonance of membrane-type acoustic metamaterials^1-3^. Therefore, the input acoustic impedance of the FAM is considered to be the acoustic impedance at the position of -*d*.

Given the one-dimensional harmonic oscillation equation in air along the *x*-direction is $\varepsilon=\varepsilon_{0}e^{i(-k_{\text{a}}x)}$, the time dependence e*^iωt^* of the sound field is assumed and canceled, where *ω* is the angular frequency of the sound, *k*_a_ is the wavenumber of sound traveling in air. The acoustic pressure (*P*_I_), particle velocity (*v*_I_) at *x* = -*d* are

$\begin{matrix} P_{\text{I}}=P_{\text{i}}+P_{\text{r}}=P_{\text{i,a}}e^{i(k_{\text{a}}d)}+P_{\text{r,a}}\text{e}^{i(-k_{\text{a}}d)} \\ v_{\text{I}}=v_{\text{i,a}}e^{i(k_{\text{a}}d)}-v_{\text{r,a}}e^{i(-k_{\text{a}}d)} \end{matrix}$ (S4)

where *P*_i,a_, *v*_i,a_ and *P*_r,a_, *v*_r,a_ are the complex amplitudes of pressure and velocity for the incident and reflected waves, respectively. They satisfy

$\begin{matrix} P_{\text{i,a}}=Z_{\text{a}}v_{\text{i,a}}S \\ P_{\text{r,a}}=Z_{\text{a}}v_{\text{r,a}}S \end{matrix}$ (S5)

where *Z*_a_ is the characteristic acoustic impendence of air. The acoustic impendence *Z*_d_ at *x* = -*d* is the ratio of the *P*_I_ and volume velocity of *v*_I_*S*, therefore,

$Z_{\text{d}}=\frac{P_{\text{I}}}{v_{\text{I}}S}$ (S6)

Substituting equation S4 and S5 to equation S6, *Z*_d_ can be obtained

$Z_{\text{d}}=\frac{P_{\text{i,a}}e^{i(k_{a}d)}+P_{\text{r,a}}e^{i(-k_{a}d)}}{\frac{P_{\text{i,a}}}{Z_{\text{a}}}e^{i(k_{a}d)}-\frac{P_{\text{r,a}}}{Z_{\text{a}}}e^{i(-k_{a}d)}}$ (S7)

We first consider that the water layer has no mass, thus there is no acoustic pressure amplitude change across the water layer. Namely,

$\frac{P_{\text{r,a}}}{P_{\text{i,a}}}=\frac{Z_{\text{w}}-Z_{\text{a}}}{Z_{\text{w}}+Z_{\text{a}}}$ (S8)

where *Z*_w_ is characteristic acoustic impendence of water. From equation S7 and S8, *Z*_d_ can be obtained as

**$Z_{\text{d}}=\frac{Z_{\text{w}}}{1+(Z_{\text{w}}^{2}/Z_{\text{a}}^{2}-1)\sin^{2}(k_{\text{a}}d)}-i\frac{Z_{\text{a}}}{2}\frac{(Z_{\text{w}}^{2}/Z_{\text{a}}^{2}-1)\sin(2k_{\text{a}}d)}{1+(Z_{\text{w}}^{2}/Z_{\text{a}}^{2}-1)\sin^{2}(k_{\text{a}}d)}$**  (S9)

where *Z*_a_ *= ρ*_a_*c*_a_ */ S* and *Z*_w_ *= ρ*_w_*c*_w_ */ S*, the *ρ*_a_, *c*_a_ and *ρ*_w_, *c*_w_, are the mass density and phase velocity of air and water, respectively. The acoustic impedance equation usually includes the acoustic damping term of *b*_m_, the acoustic mass term of *ωM*_m_, and the acoustic compliance term of *K*_m_/*ω* (Ref. 3). There is not a mass term in equation S9 because we didn’t consider the mass. Since *h* and *d* are both much smaller than the operating wavelength of sound, the system in Scheme S2 can be treated as a lumped-parameter system, therefore the mass of the water layer can be inserted to the equation S9 (Ref. 1). Namely, given the water layer has a thickness of *h*, the acoustic impendence can be obtained as

$Z_{\text{d}}=\frac{Z_{\text{w}}}{1+(Z_{\text{w}}^{2}/Z_{\text{a}}^{2}-1)\sin^{2}(k_{\text{a}}d)}+i(\frac{k_{\text{a}}h\rho_{\text{w}}c_{\text{a}}}{S}-\frac{Z_{\text{a}}}{2}\frac{(Z_{\text{w}}^{2}/Z_{\text{a}}^{2}-1)\sin(2k_{\text{a}}d)}{1+(Z_{\text{w}}^{2}/Z_{\text{a}}^{2}-1)\sin^{2}(k_{\text{a}}d)})$ (S10)

where the acoustic damping, acoustic mass, and acoustic compliance terms are:

$\begin{matrix} b_{\text{m}}=\frac{Z_{\text{w}}}{1+(Z_{\text{w}}^{2}/Z_{\text{a}}^{2}-1)\sin^{2}(k_{\text{a}}d)} \\ \omega M_{\text{m}}=\frac{k_{\text{a}}c_{\text{a}}h\rho_{\text{w}}}{S} \\ \frac{K_{\text{m}}}{\omega}=\frac{Z_{\text{a}}}{2}\frac{(Z_{\text{w}}^{2}/Z_{\text{a}}^{2}-1)\sin(2k_{\text{a}}d)}{1+(Z_{\text{w}}^{2}/Z_{\text{a}}^{2}-1)\sin^{2}(k_{\text{a}}d)}) \end{matrix}$ (S11)

1. **The impedance matched surface**

The reflection coefficient at *x* = -*d* in scheme S2 is

$R=\frac{Z_{\text{d}}-Z_{\text{a}}}{Z_{\text{d}}+Z_{\text{a}}}$ (S12)

The reflection coefficient equals zero for the total transmission, namely, *Z*_d_ = *Z*_a_. Namely,

$\omega M_{\text{m}}-K_{\text{m}}/\omega=0$ (S13)

$b_{\text{m}}=Z_{\text{a}}$ (S14)

Since the discussion above doesn’t consider the dissipation from thermoviscous losses, the no reflection means the unit transmission. Equation S13 suggests that the frequency of unity transmission equals the natural resonant frequency. Because sin*k*_a_*d* ≈ *k*_a_*d* and Z_w_ >> Z_a_, the frequency of unity transmission (*f*_u_) can be obtained with equation S11 and S13.

$f_{\text{u}}=\frac{1}{2\pi}\sqrt{\frac{K_{\text{m}}}{M_{\text{m}}}}\approx\frac{c_{\text{a}}}{2\pi}\sqrt{\frac{\rho_{\text{a}}}{hd\rho_{\text{w}}}}$ (S15)

From equation S14 and S15, we can get the following equations:

$d\approx\frac{c_{\text{a}}}{2\pi f_{\text{u}}}\sqrt{\frac{\rho_{\text{a}}c_{\text{a}}}{\rho_{\text{w}}c_{\text{w}}}}$ (S16)

$h\approx\frac{1}{2\pi f_{\text{u}}\rho_{\text{w}}}\sqrt{\rho_{\text{a}}c_{\text{a}}\rho_{\text{w}}c_{\text{w}}}$ (S17)

$\frac{h}{d}\approx\frac{c_{\text{w}}}{c_{\text{a}}}$ (S18)

From equation S15-S18, the following conclusions can be obtained. (**1**) The frequency of unity transmission *f*_u_ corresponds to a certain value of *h* and *d* and is determined by *hd*. (**2**) The precondition for the existence of *f*_u_ is *h*/*d* = *c*_w_/*c*_a_. In other words, the *h*/*d* = *c*_w_/*c*_a_ is an impedance matching condition (IMC). Once it is satisfied, it must exist a frequency of unity transmission. (**3**) The unity transmission only occurs at the natural resonant frequency of FAM.

1. **The mass-spring model for the ideal FAM**

When the resonance occurs, the mass *m*, sprig constant *k*, and the damping coefficient *b* can be calculated by substituting the resonant frequency (equation S15) to equation S11.

$$k=K_{\text{m}}S^{2}=\frac{\rho_{\text{a}}c_{\text{a}}^{2}}{d}S$$

$$m=M_{\text{m}}S^{2}=h\rho_{\text{w}}S$$

$b=b_{\text{m}}S^{2}=\rho_{\text{a}}c_{\text{a}}S$ (S19)

The quality factor, *Q*, can be deduced from equation above.

$Q=\frac{\sqrt{mk}}{b}=\sqrt{\frac{\rho_{\text{w}}h}{\rho_{\text{a}}d}}=\frac{1}{k_{a}d}$ (S20)

When *h*/*d* = *c*_w_/*c*_a_,

$Q=\sqrt{\frac{\rho_{\text{w}}h}{\rho_{\text{a}}d}}=\sqrt{\frac{\rho_{\text{w}}c_{\text{w}}}{\rho_{\text{a}}c_{\text{a}}}}\approx\sqrt{\frac{998\times1480}{1.21\times344}}=59.57$ (S21)

**Note S3**

**The analytical model for calculating the transmission coefficients**


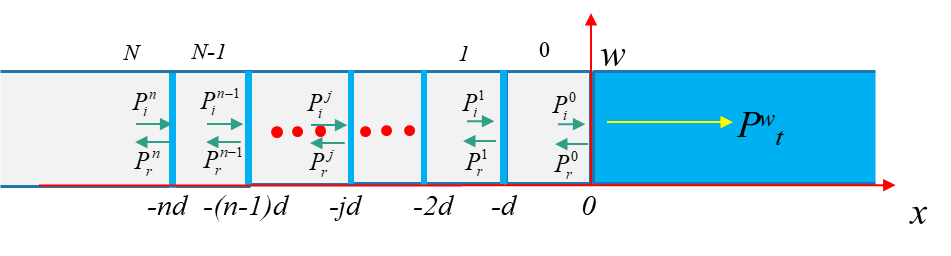


**Scheme S3**. Schematic diagram of the reflection and transmission on the FAMs with *N* layers of air and water. The *N* layers of air divide the space into *N*+1 spaces, as marked with *0, 1,***…***,N-1, N*. Positions of the air-water interfaces are marked with 0, -*d*, -2*d*, **…,** -*jd,* **…, -***nd*. The $P_{i}^{j}$, $P_{r}^{j}$ are the acoustic pressure amplitudes of the incident and reflected waves at the air-water interface of *j*. The $P_{t}^{w}$ is the acoustic pressure amplitudes of the transmitted waves.

The IMC calculations in Note S2 predicts the frequency of unity transmission when *h*/*d* = *c*_w_/*c*_a_. But it cannot work for the more general situation that *h*/*d* ≠ *c*_w_/*c*_a_. Next we conduct an analytical model to calculate the transmission curves for any frequency and any ratio of *h* and *d*. We will directly deduce the transmission curves for arbitrary finite *N* layer ideal FAM (without considering the dissipation). The one layer, namely, *N* = 1, will be applied to confirm the conclusions from the IMC calculations in Note S2. The multilayer (*N* > 1) will be used to confirm the predictions of the multilayer FAMs in Fig. 3 and Note S6.

Given the water layers in Scheme S3 having the identical thickness of *h*, the water layer is also considered to oscillate integrally and the sound transmitting within the water layer is not considered. The acoustic pressure and particle velocity at different positions are marked with *P*(*x*) and *v*(*x*).

At the space marked with *N*, namely, the *N* space, the position is *x* < -*nd*

$\begin{matrix} P^{N}(x)=P_{\text{i}}^{N}e^{i(-k_{\text{a}}x)}+P_{\text{r}}^{N}e^{i(k_{\text{a}}x)} \\ u^{N}(x)=\frac{P_{\text{i}}^{N}e^{i(-k_{\text{a}}x)}}{SZ_{\text{a}}}-\frac{P_{\text{r}}^{N}e^{i(k_{\text{a}}x)}}{SZ_{\text{a}}} \end{matrix}$ (S22)

At the *J* space (1≤ *J* ≤ *N*) namely, the position of – (*j*+1)*d* < *x* < -*jd,* where 1 ≤ *j* ≤ *n,*

$\begin{matrix} P^{J}(x)=P_{\text{i}}^{J}e^{i(-k_{\text{a}}x)}+P_{\text{r}}^{J}e^{i(k_{\text{a}}x)} \\ u^{J}(x)=\frac{P_{\text{i}}^{J}e^{i(-k_{\text{a}}x)}}{SZ_{\text{a}}}-\frac{P_{\text{r}}^{J}e^{i(k_{\text{a}}x)}}{SZ_{\text{a}}} \end{matrix}$ (S23)

At the *0* space, namely, the position of –*d* < *x* < 0,

$\begin{matrix} P^{0}(x)=P_{\text{i}}^{0}e^{i(-k_{\text{a}}x)}+P_{\text{r}}^{0}e^{i(k_{\text{a}}x)} \\ u^{0}(x)=\frac{P_{\text{i}}^{0}e^{i(-k_{\text{a}}x)}}{SZ_{\text{a}}}-\frac{P_{\text{r}}^{0}e^{i(k_{\text{a}}x)}}{SZ_{\text{a}}} \end{matrix}$ (S24)

For the bulk water, namely, the position of *x* > 0

$\begin{matrix} P^{\text{w}}(x)=P_{\text{t}}^{\text{w}}e^{i(-k_{\text{w}}x)} \\ u^{\text{w}}(x)=\frac{P_{\text{t}}^{\text{w}}e^{i(-k_{\text{w}}x)}}{SZ_{\text{w}}} \end{matrix}$ (S25)

where *k*_w_ is the wavenumber of sound traveling in water.

If the reflection and transmission coefficient at the air-water interface of *j* are marked with *R*_j_ and *T*_j_. Combining with equation S22-S25, they can be calculated as follows:

At the interface of *x* = *-nd*,

$R_{n}=\frac{\left.P_{\text{r}}^{N}e^{i(k_{\text{a}}x)}\right|_{x=-nd}}{\left.P_{\text{i}}^{N}e^{i(-k_{\text{a}}x)}\right|_{x=-nd}}=\frac{P_{\text{r}}^{N}}{P_{\text{i}}^{N}e^{i2nk_{\text{a}}d}}$ $T_{n}=\frac{\left.P_{\text{i}}^{N-1}e^{i(-k_{\text{a}}x)}\right|_{x=-nd}}{\left.P_{\text{i}}^{N}e^{i(-k_{\text{a}}x)}\right|_{x=-nd}}=\frac{P_{\text{i}}^{N-1}}{P_{\text{i}}^{N}}$ (S26)

At the interface of *x* = *-jd*, where 1 ≤ *j* ≤ *n*,

$R_{j}=\frac{\left.P_{\text{r}}^{j}e^{i(k_{\text{a}}x)}\right|_{x=-jd}}{\left.P_{\text{i}}^{j}e^{i(-k_{\text{a}}x)}\right|_{x=-jd}}=\frac{P_{\text{r}}^{j}}{P_{\text{i}}^{j}e^{i2jk_{\text{a}}d}}$ $T_{j}=\frac{\left.P_{\text{i}}^{j-1}e^{i(-k_{\text{a}}x)}\right|_{x=-jd}}{\left.P_{\text{i}}^{j}e^{i(-k_{\text{a}}x)}\right|_{x=-jd}}=\frac{P_{\text{i}}^{j-1}}{P_{\text{i}}^{j}}$ (S27)

At the interface of *x* = 0,

**$R_{0}=\frac{\left.P_{\text{r}}^{0}e^{i(k_{\text{a}}x)}\right|_{x=0}}{\left.P_{\text{i}}^{0}e^{i(-k_{\text{a}}x)}\right|_{x=0}}=\frac{P_{\text{r}}^{0}}{P_{\text{i}}^{0}}$**  (S28)

The total transmission coefficient *T* can be shown as:

**$T=\frac{\left.P_{\text{t}}^{\text{w}}e^{i(k_{\text{w}}x)}\right|_{x=0}}{\left.P_{\text{i}}^{N}e^{i(-k_{\text{a}}x)}\right|_{x=-nd}}=\frac{P_{\text{t}}^{\text{w}}}{P_{\text{i}}^{N}e^{ink_{\text{a}}d}}$**  (S29)

Next, we will deduce total transmission coefficient *T* with the boundary conditions at different air-water interfaces. At the air-water interface of *x* = 0:

$p_{\text{i}}^{0}\text{+ }p_{\text{r}}^{0}=p_{\text{t}}^{\text{w}}$ (S30)

$\left.u^{0}\right|_{x=0}=\left.u^{\text{w}}\right|_{x=0}$ (S31)

Substituting the equation S28, S29 to equation S30,

$1+R_{0}=\frac{P_{\text{t}}^{\text{w}}}{P_{\text{i}}^{0}}=\frac{P_{\text{t}}^{N}}{P_{\text{i}}^{0}}e^{ink_{\text{a}}d}$ (S32)

From equation S27-S29,

$\frac{P_{\text{t}}^{N}}{P_{\text{i}}^{0}}=\frac{P_{\text{t}}^{N}}{P_{\text{i}}^{N-1}}\times\frac{P_{\text{t}}^{N-1}}{P_{\text{i}}^{N-2}}\cdots\times\frac{P_{\text{t}}^{1}}{P_{\text{i}}^{0}}=\frac{1}{\prod_{q=1}^{n} T_{q}}$ (S33)

Combining the equation S32 and S33,

$1+R_{0}=\frac{Te^{ink_{\text{a}}d}}{\prod_{q=1}^{n} T_{q}}$ (S34)

Similarly, from equations of S31, S24 and S25, we can get

$1-R_{0}=\frac{Z_{\text{a}}}{Z_{\text{w}}}\frac{Te^{ink_{\text{a}}d}}{\prod_{q=1}^{n} T_{q}}$ (S35)

Combining equation of S34 and S35, *R*_0_ and *T* can be obtained:

$R_{0}=\frac{Z_{\text{w}}-Z_{\text{a}}}{Z_{\text{w}}+Z_{\text{a}}}$ (S36)

$T=2\frac{Z_{\text{w}}}{Z_{\text{w}}+Z_{\text{a}}}e^{-ink_{\text{a}}d}\prod_{q=1}^{n} T_{q}$ (S37)

The equation S37 suggests that to calculate the total transmission coefficient *T*, the transmission coefficient at every air-water interface should be calculated firstly.

As shown in Scheme S3, at the air-water interface of *j*, namely, *x = -jd*, where 1 ≤ *j* ≤ *n*, the boundary conditions are:

*$(\left.P^{J}\right|_{x=-jd}\left.-P^{J-1}\right|_{x=-jd})S=m_{j}\frac{\left.du^{J}\right|_{x=-jd}}{dt}=i\omega m_{j}\left.u^{J}\right|_{x=-jd}$*

$\left.u^{J}\right|_{x=-jd}=\left.u^{J-1}\right|_{x=-jd}$ （S38）

where *m*_j_ is the mass of the *j*th water layer. Given that each water layer has the same thickness of *h*, *m*_j_ = *ρ*_w_*Sh*. Similar with the derivation of equation S34 and S35, the following equations can be obtained from S38 and S27.

$1+R_{j}=T_{j}[i\omega\frac{\rho_{\text{w}}h}{SZ_{\text{a}}}(1-R_{j-1}e^{-i2k_{\text{a}}d})+(1+R_{j-1}e^{-i2k_{\text{a}}d})]$ (S39)

$1-R_{j}=T_{j}(1-R_{j-1}e^{-i2k_{\text{a}}d})$ (S40)

The transmission coefficient at each air-water interface can be obtained for the above two equations.

$T_{j}=\frac{2}{2+i\omega\frac{\rho_{\text{w}}h}{SZ_{\text{a}}}(1-R_{j-1}e^{-i2k_{\text{a}}d})}$ (S41)

The equation S41 gives the relation of the transmission coefficient (*T_j_*) at the air-water interface of *j* and the reflection coefficient (*R_j_*_-1_) at the air-water interface of *j*-1. Equation S40 shows the relation between *T_j_* and *R_j_*. Therefore, the transmission and reflection coefficient at every water-air interface can be obtained with these two equations. For example, when *j* =1, from equation S41, we can obtain *T*_1_ with *R*_0_ that is form equation S40. Then we can get *R*_1_ with *T*_1_ and *R*_0_ with equation S40, and *T*_2_ can be obtained from *R*_1_ with equation S41. With these processes, again and again, the transmission coefficient at every air-water interface can be achieved. The processes are shown as follows:


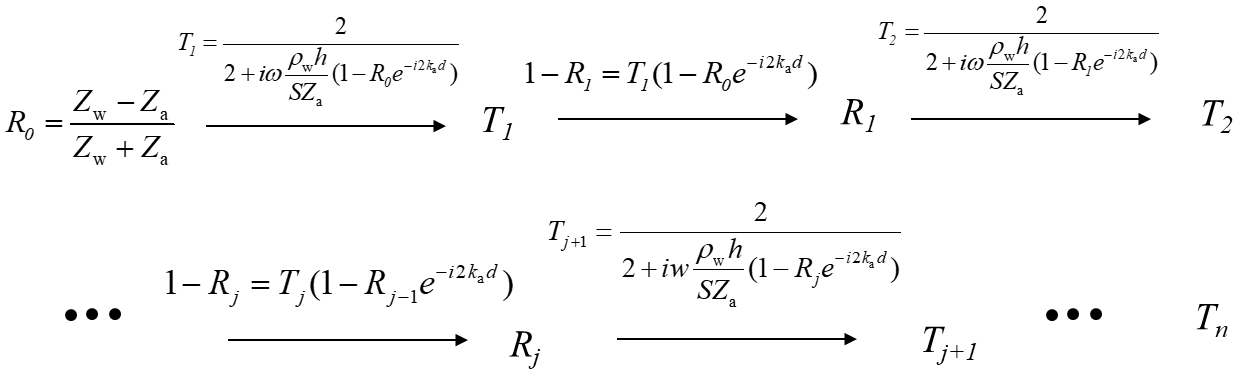


Then with the equation S37, we can achieve the total transmission coefficient *T*. The analytical expressions for three and more layers of water are very complex. We have obtained the analytical expression for the one (*T_N_*_=1_) and two layers (*T_N_*_=2_):

$T_{N=1}=\frac{2\frac{Z_{\text{w}}}{Z_{\text{a}}}}{(1+\frac{Z_{\text{w}}}{Z_{\text{a}}})\cos k_{\text{a}}d-\frac{\omega\rho_{\text{w}}hZ_{\text{w}}}{SZ_{\text{a}}^{2}}\sin k_{\text{a}}d+i[(1+\frac{Z_{\text{w}}}{Z_{\text{a}}})\sin k_{\text{a}}d+\frac{\omega\rho_{\text{w}}h}{SZ_{\text{a}}}\cos k_{\text{a}}d]}$ (S42)

$T_{N=2}=\frac{4\frac{Z_{\text{w}}}{Z_{\text{a}}}}{[2(\frac{Z_{\text{w}}}{Z_{\text{a}}}+1)-\frac{Z_{\text{w}}}{Z_{\text{a}}}A^{2}]\cos2k_{\text{a}}d-A(\frac{3Z_{\text{w}}}{Z_{\text{a}}}+1)\sin2k_{\text{a}}d+\frac{Z_{\text{w}}}{Z_{\text{a}}}A^{2}+i[(2(\frac{Z_{\text{w}}}{Z_{\text{a}}}+1)-A^{2})\sin2k_{\text{a}}d+A(\frac{Z_{\text{w}}}{Z_{\text{a}}}+3)\cos2k_{\text{a}}d-A(\frac{Z_{\text{w}}}{Z_{\text{a}}}-1)]}$ (S43)

where $A=\frac{\omega\rho_{\text{w}}h}{Z_{\text{a}}S}$, $Z_{\text{w}}=\rho_{\text{w}}c_{\text{w}}/S$, $Z_{\text{a}}=\rho_{\text{a}}c_{\text{a}}/S$, and $\omega=k_{\text{a}}c_{\text{a}}$.

The energy transmission coefficient *τ* can be obtained from equation S42 for *N*=1.

$\tau=\frac{Z_{\text{a}}}{Z_{\text{w}}}\left|T_{N\text{=1}}\right|^{2}=\frac{\frac{4Z_{\text{w}}}{Z_{\text{a}}}}{[(1+\frac{Z_{\text{w}}}{Z_{\text{a}}})\cos k_{\text{a}}d-\frac{\omega\rho_{\text{w}}hZ_{w}}{SZ_{\text{a}}^{2}}\sin k_{\text{a}}d]^{2}+[(1+\frac{Z_{\text{w}}}{Z_{\text{a}}})\sin k_{\text{a}}d+\frac{\omega\rho_{\text{w}}h}{SZ_{\text{a}}}\cos k_{\text{a}}d]^{2}}$ (S44)

Similarly, the reflection coefficient *R* and energy reflection coefficient *r* can be obtained with equation S34 and S42,

$$R=\frac{(\frac{Z_{\text{w}}}{Z_{\text{a}}}-1)\cos k_{\text{a}}d-\frac{\omega\rho_{\text{w}}hZ_{\text{w}}}{SZ_{\text{a}}^{2}}\sin k_{\text{a}}d\text{+}i[(1-\frac{Z_{\text{w}}}{Z_{\text{a}}})\sin k_{\text{a}}d+\frac{\omega\rho_{\text{w}}h}{SZ_{\text{a}}}\cos k_{\text{a}}d]}{(1+\frac{Z_{\text{w}}}{Z_{\text{a}}})\cos k_{\text{a}}d-\frac{\omega\rho_{\text{w}}hZ_{w}}{SZ_{\text{a}}^{2}}\sin k_{\text{a}}d+i[(1+\frac{Z_{\text{w}}}{Z_{\text{a}}})\sin k_{\text{a}}d+\frac{\omega\rho_{\text{w}}h}{SZ_{\text{a}}}\cos k_{\text{a}}d]}$$

$r=R^{2}=\frac{[(\frac{Z_{\text{w}}}{Z_{\text{a}}}-1)\cos k_{\text{a}}d-\frac{\omega\rho_{\text{w}}hZ_{\text{w}}}{SZ_{\text{a}}^{2}}\sin k_{\text{a}}d]^{2}\text{+}[(1-\frac{Z_{\text{w}}}{Z_{\text{a}}})\sin k_{\text{a}}d+\frac{\omega\rho_{\text{w}}h}{SZ_{\text{a}}}\cos k_{\text{a}}d]^{2}}{[(1+\frac{Z_{\text{w}}}{Z_{\text{a}}})\cos k_{\text{a}}d-\frac{\omega\rho_{\text{w}}hZ_{\text{w}}}{SZ_{\text{a}}^{2}}\sin k_{\text{a}}d]^{2}+[(1+\frac{Z_{\text{w}}}{Z_{\text{a}}})\sin k_{\text{a}}d+\frac{\omega\rho_{\text{w}}h}{SZ_{\text{a}}}\cos k_{\text{a}}d]^{2}}$ (S45)

**Note S4**

**The analytical model agrees very well with calculations from impedance matching conditions.**

The frequency with the maximum energy transmission coefficient (marked with *f*_max_) can be calculated by taking a derivation of frequency for equation S44. Namely,

$\frac{\partial\tau}{\partial f}\text{=}0$ (S46)

With the conditions that *k*_a_*d* << 1, sin*k*_a_*d* ≈ *k*_a_*d* and *Z*_w_ >> *Z*_a_, we can get

$f_{\text{max}}\approx\frac{c_{\text{a}}}{2\pi}\sqrt{\frac{\rho_{\text{a}}}{hd\rho_{\text{w}}}}$ (S47)

Namely, the maximum energy transmission (*τ*_max_) occurs at the resonant frequency. By substituting equation S47 into equation S44, we can get

$\tau_{\text{max}}=\frac{\frac{4Z_{\text{w}}}{Z_{\text{a}}}}{1+[(1+\frac{Z_{\text{w}}}{Z_{\text{a}}})\sqrt{\frac{\rho_{\text{a}}d}{\rho_{\text{w}}h}}+\sqrt{\frac{\rho_{\text{w}}h}{\rho_{\text{a}}d}}]^{2}}\approx\frac{\frac{4c_{\text{w}}}{c_{\text{a}}}}{(\frac{c_{\text{w}}}{c_{\text{a}}}\sqrt{\frac{d}{h}}+\sqrt{\frac{h}{d}})^{2}}$ (S48)

The equation above suggests the *τ*_max_ depends on the ratio of *h* and *d*. The maximum of the *τ*_max_ occurs when

$\frac{\partial\tau_{\max}}{\partial(h/d)}\text{=}0$ (S49)

From equation S48-S49 we can get that the maximum of *τ*_max_ equals 1 when *h*/*d*=*c*_w_/*c*_a_.

The discussion above gives the same conclusions with the IMC calculations. It suggests, when *h*/*d* = *c*_w_/*c*_a_, the maximum transmission frequency (*f*_max_) is the frequency of unity transmission (*f*_u_). In summary, for the ideal FAM, the maximum energy transmission coefficient always occurs at its resonant frequency (*f*_R_). The maximum transmission coefficient is determined by *h*/*d*, only when *h*/*d* = *c*_w_/*c*_a_, the unity transmission occurs, namely,

$f_{\text{max}}=f_{\text{R}}=f_{\text{u}}$ (S50)

**Note S5**

**The effect of the solid structure**

The existence of the solid structure will have a large influence on the maximum transmission frequency because it reduces the volume of the air layer. As shown in Scheme S4, the cross-sectional area has shrined to *S*_d_ from *S*, which changes the acoustic impedance according to equation S6.


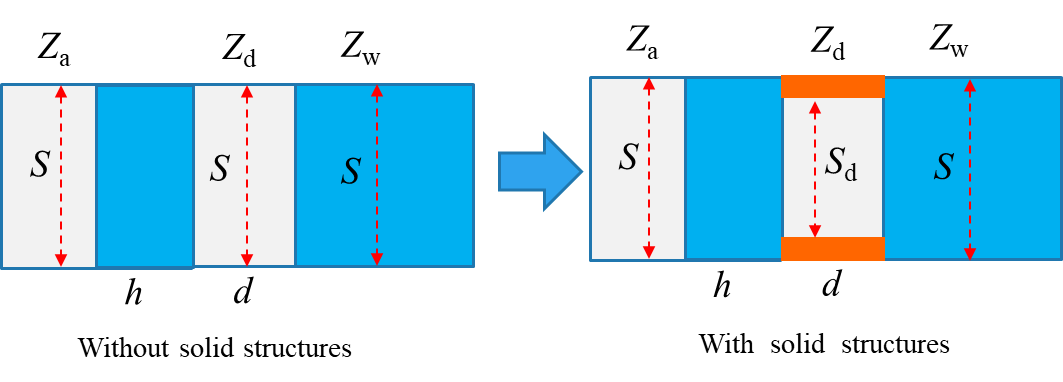


Scheme S4. The geometry of the model with and without solid structures.

The *β* is defined to exhibit the cross-sectional area change, namely,

$\beta\equiv\frac{S_{\text{d}}}{S}$ (S51)

In the calculations in Note S2, the equation S8 changes to

$\frac{P_{\text{r,a}}}{P_{\text{i,a}}}=\frac{\frac{\rho_{\text{w}}c_{\text{w}}}{S}-\frac{\rho_{\text{a}}c_{\text{a}}}{S_{\text{d}}}}{\frac{\rho_{\text{w}}c_{\text{w}}}{S}+\frac{\rho_{\text{a}}c_{\text{a}}}{S_{\text{d}}}}=\frac{\beta\frac{\rho_{\text{w}}c_{\text{w}}}{S}-\frac{\rho_{\text{a}}c_{\text{a}}}{S}}{\beta\frac{\rho_{\text{w}}c_{\text{w}}}{S}+\frac{\rho_{\text{a}}c_{\text{a}}}{S}}=\frac{\beta Z_{\text{w}}-Z_{\text{a}}}{\beta Z_{\text{w}}+Z_{\text{a}}}$ (S52)

The equation S6 changes to

$Z_{\text{d}}=\frac{P_{\text{I}}}{v_{\text{I}}S_{\text{d}}}$ (S53)

Therefore, the equation S7 will be

$Z_{\text{d}}=\frac{P_{\text{i,a}}e^{-ik_{a}d}+P_{\text{r,a}}e^{ik_{a}d}}{\frac{P_{i,a}S_{\text{d}}}{Z_{\text{a}}S}e^{-ik_{a}d}-\frac{P_{r,a}S_{\text{d}}}{Z_{\text{a}}S}e^{ik_{a}d}}=\frac{P_{\text{i,a}}e^{-ik_{a}d}+P_{\text{r,a}}e^{ik_{a}d}}{\beta(\frac{P_{i,a}}{Z_{\text{a}}}e^{-ik_{a}d}-\frac{P_{r,a}}{Z_{\text{a}}}e^{ik_{a}d})}$ (S54)

Substituting equation S52 to S54, and inserting the acoustic mass term, equation S10 changes to

$Z_{\text{d}}=\frac{Z_{\text{w}}}{1+(\beta^{2}Z_{\text{w}}^{2}/Z_{\text{a}}^{2}-1)\sin^{2}(k_{\text{a}}d)}+i(\frac{k_{\text{a}}h\rho_{\text{w}}c_{\text{a}}}{S}-\frac{Z_{\text{a}}}{2\beta}\frac{(\beta^{2}Z_{\text{w}}^{2}/Z_{\text{a}}^{2}-1)\sin(2k_{\text{a}}d)}{1+(\beta^{2}Z_{\text{w}}^{2}/Z_{\text{a}}^{2}-1)\sin^{2}(k_{\text{a}}d)})$ (S55)

With the impedance matched condition that *Z*_d_ = *Z*_a_ = *ρ*_a_*c*_a_/*S*, the following results will be obtained

$f_{\text{u}}=\frac{c_{\text{a}}}{2\pi}\sqrt{\frac{\rho_{\text{a}}}{h\beta d\rho_{\text{w}}}}$ $\beta d\approx\frac{c_{\text{a}}}{2\pi f_{\text{u}}}\sqrt{\frac{\rho_{\text{a}}c_{\text{a}}}{\rho_{\text{w}}c_{\text{w}}}}$

$h\approx\frac{1}{2\pi f_{\text{u}}\rho_{\text{w}}}\sqrt{\rho_{\text{a}}c_{\text{a}}\rho_{\text{w}}c_{\text{w}}}$ $\frac{h}{\beta d}\approx\frac{c_{\text{w}}}{c_{\text{a}}}$ (S56)

The mass *m*, sprig constant *k* and the damping coefficient *b*, quality factor *Q* changes to

$k=\frac{\rho_{\text{a}}c_{\text{a}}^{2}}{\beta d}S$ $m=h\rho_{\text{w}}S$ $b=\rho_{\text{a}}c_{\text{a}}S$ $Q=\frac{1}{k_{\text{a}}\beta d}$ (S57)

If the *βd* in equation S56 changes to *d*, namely, *β =* 1, the equations S56 are the same with equation S15-S18. Therefore, the existence of the solid structure reduces the air layer thickness from *d* to *βd*. With the similar processes, the energy transmission and reflection coefficients for *N* = 1 can be obtained

$$\tau=\frac{\frac{4Z_{\text{w}}}{Z_{\text{a}}}}{[(1+\frac{Z_{\text{w}}}{Z_{\text{a}}})\cos k_{\text{a}}d-\frac{\omega\rho_{\text{w}}hZ_{w}}{SZ_{\text{a}}^{2}}\beta\sin k_{\text{a}}d]^{2}+[(\frac{1}{\beta}+\beta\frac{Z_{\text{w}}}{Z_{\text{a}}})\sin k_{\text{a}}d+\frac{\omega\rho_{\text{w}}h}{SZ_{\text{a}}}\cos k_{\text{a}}d]^{2}}$$

$r=\frac{[(\frac{Z_{\text{w}}}{Z_{\text{a}}}-1)\cos k_{\text{a}}d-\frac{\omega\rho_{\text{w}}hZ_{\text{w}}}{SZ_{\text{a}}^{2}}\beta\sin k_{\text{a}}d]^{2}\text{+}[(\frac{1}{\beta}-\beta\frac{Z_{\text{w}}}{Z_{\text{a}}})\sin k_{\text{a}}d+\frac{\omega\rho_{\text{w}}h}{SZ_{\text{a}}}\cos k_{\text{a}}d]^{2}}{[(1+\frac{Z_{\text{w}}}{Z_{\text{a}}})\cos k_{\text{a}}d-\frac{\omega\rho_{\text{w}}hZ_{\text{w}}}{SZ_{\text{a}}^{2}}\beta\sin k_{\text{a}}d]^{2}+[(\frac{1}{\beta}+\beta\frac{Z_{\text{w}}}{Z_{\text{a}}})\sin k_{\text{a}}d+\frac{\omega\rho_{\text{w}}h}{SZ_{\text{a}}}\cos k_{\text{a}}d]^{2}}$ (S58)

Substituting the equations that $\beta=\frac{(a-w)^{2}}{a^{2}}$ into equation S56 and S58, the effect of *h*, *d*, *a*, *w* on the maximum transmission frequency of *f*_max_ and the corresponding energy transmission coefficient of *τ*_max_ can be obtained, as shown in Fig. S3.

**Note S6**

**The multilayer FAMs: series mass-spring model**

**
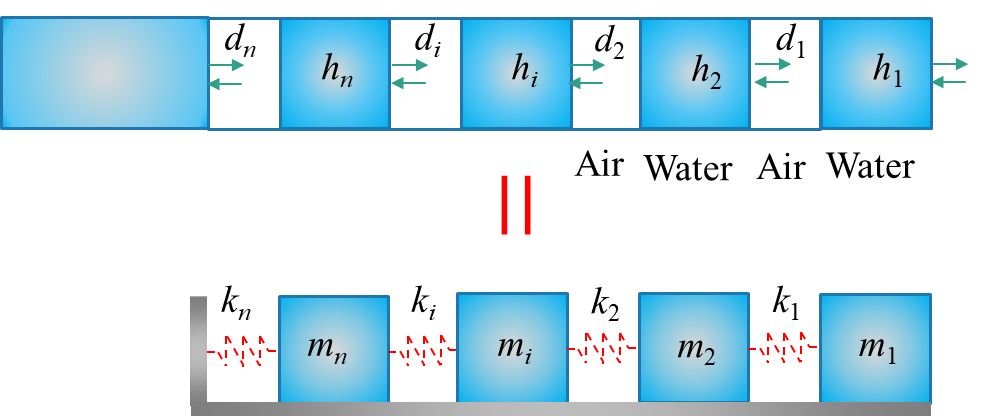
**

**Scheme S5.** Schematic diagram of the multilayer FAMs and the corresponding series mass- spring model.

The FAM consisting of one air layer and one water layer corresponds to the single degree freedom mechanical vibration^4^. The multilayer FAMs corresponds to the multi-degree freedom mechanical vibration^4,5^. As shown in Scheme S5, the series mass-spring model has many natural resonant frequencies, and all of them are the maximum transmission frequencies. Next, we will calculate these resonant frequencies with the mechanical vibration theory for the multi-degree of freedom. Considering the simplest condition that all the water and air layer are respectively the identical and no damping exists. Namely,

$\begin{matrix} m_{1}=m_{2}=m_{i}=m_{n}=\rho_{\text{w}}hS \\ k_{1}=k_{2}=k_{i}=k_{n}=\frac{\rho_{\text{a}}c_{\text{a}}^{2}}{d\beta}S \end{matrix}$ (S59)

The angular resonant frequency (*ω*_0_) for one-layer FAM is

$\omega_{0}=\sqrt{\frac{k}{m}}=\sqrt{\frac{\rho_{\text{a}}c_{\text{a}}^{2}}{hd\beta\rho_{\text{w}}}}$ (S60)

Next, we will demonstrate that the *n*-layer FAMs will have *n* angular resonant transmission frequencies, and all of them will be between 0 and 2*ω*_0_.

The free vibration equations of the series mass-spring model are^4^


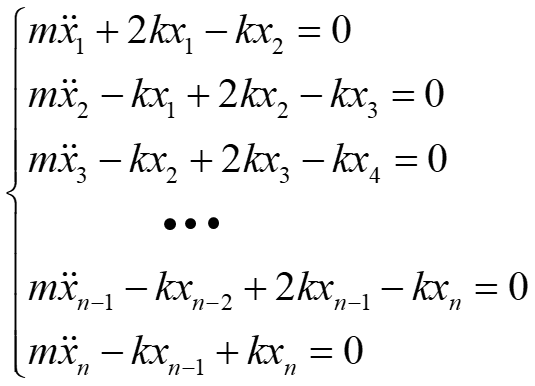
 (S61)

where *x* is the displacement of the mass. The mass matrix *M* and the spring constant matrix *K* in equation S61 are

$M\text{=}m\times\left[\begin{matrix} 1 & 0 & 0 & \cdots& 0 \\ 0 & 1 & 0 & \cdots& 0 \\ 0 & 0 & 1 & \cdots& 0 \\ \vdots& \vdots& \vdots& \ddots& 0 \\ 0 & 0 & 0 & \cdots& 1 \end{matrix}\right]_{n}=mC$ $K=k\times\left[\begin{matrix} 2 & -1 & 0 & 0 & \cdots& 0 & 0 & 0 \\ -1 & 2 & -1 & 0 & \cdots& 0 & 0 & 0 \\ 0 & -1 & 2 & -1 & \cdots& 0 & 0 & 0 \\ \vdots& \vdots& \vdots& \vdots& \ddots& \vdots& \vdots& \vdots\\ 0 & 0 & 0 & 0 & \cdots& -1 & 2 & -1 \\ 0 & 0 & 0 & 0 & \cdots& 0 & -1 & 1 \end{matrix}\right]_{n}=kD$ (S62)

The matrix *C* and matric *D* are defined as shown in equation S62. And the equation S61 can be written as

$(K-\omega^{2}M)\phi=0$ (S63)

where *φ* is the vector of amplitudes of *x*, substituting equation S60, S62 to S63,

$(\omega_{0}^{2}D-\omega^{2}C)\phi=0$ (S64)

The characteristic equation of the equation S64 can be transferred to

$\left|\begin{matrix} 2-\frac{\omega^{2}}{\omega_{0}^{2}} & -1 & 0 & 0 & \cdots& 0 & 0 & 0 \\ -1 & 2-\frac{\omega^{2}}{\omega_{0}^{2}} & -1 & 0 & \cdots& 0 & 0 & 0 \\ 0 & -1 & 2-\frac{\omega^{2}}{\omega_{0}^{2}} & -1 & \cdots& 0 & 0 & 0 \\ \vdots& \vdots& \vdots& \vdots& \ddots& \vdots& \vdots& \vdots\\ 0 & 0 & 0 & 0 & \cdots& -1 & 2-\frac{\omega^{2}}{\omega_{0}^{2}} & -1 \\ 0 & 0 & 0 & 0 & \cdots& 0 & -1 & 1-\frac{\omega^{2}}{\omega_{0}^{2}} \end{matrix}\right|_{n}=0$ (S65)

Marking that the left term above is *Z*_n_, we can get that, for *n* ≥ 4

$\text{Z}_{n}=(2-\frac{\omega^{2}}{\omega_{0}^{2}})\times Z_{n-1}-Z_{n-2}$ (S66)

Defining a parameter of *α* satisfying that

$\alpha+\frac{1}{\alpha}=2-\frac{\omega^{2}}{\omega_{0}^{2}}$ (S67)

The following equation can be obtained with equation S66 and S67

$Z_{n}=\frac{(\alpha Z_{3}-Z_{2})\alpha^{n-2}-(Z_{3}-\alpha Z_{2})\alpha^{3-n}}{\alpha^{2}-1}$ (S68)

where

$\begin{matrix} Z_{2}=(\frac{\omega^{2}}{\omega_{0}^{2}})^{2}-3(\frac{\omega^{2}}{\omega_{0}^{2}})+1 \\ Z_{3}=-(\frac{\omega^{2}}{\omega_{0}^{2}})^{3}+5(\frac{\omega^{2}}{\omega_{0}^{2}})^{2}-6(\frac{\omega^{2}}{\omega_{0}^{2}})+1 \end{matrix}$ (S69)

Substituting the equation S69 and S67 into S68, *Z*_n_ can be obtained

$Z_{n}=\frac{\alpha^{n+1}+\alpha^{-n}}{\alpha+1}$ (S70)

Combining with that *Z*_n_ is zero from equation S65, we can get that

$\frac{\alpha^{2n+1}+1}{\alpha+1}\text{=}0$ (S71)

From equation S67

$\omega=\omega_{0}\sqrt{(2-\alpha-\frac{1}{\alpha})}$ (S72)

where *n* ≥ 1. The equation S71 has 2*n* complex roots and they include *n* complex conjugates. Substituting the roots into equation S72, the *ω* will have *n* real values. Therefore, the equation S71 and S72 will give *n* resonant angular frequency. The following gives the demonstration.

Given that the 2*n* roots in equation S71 have the form that

$\alpha=q\pm si$ (S73)

where *q* and *s* are both real numbers. Therefore,

$2-\alpha-\frac{1}{\alpha}=2-(q\pm si+\frac{q\mp si}{q^{2}+s^{2}})=2-q(1+\frac{1}{q^{2}+s^{2}})\mp s(1-\frac{1}{q^{2}+s^{2}})i$ (S74)

The solutions of equation S65 are real^2^. Namely, the value of equation S74 is real, we can get

$q^{2}+s^{2}=1$ (S75)

Because *q* and *s* are real, namely,

-1 ≤ *q* ≤ 1 (S76)

From S72 and S74,

$\omega=\omega_{0}\sqrt{2(1-q)}$ (S77)

From equation S76 and S77, we can get

$0\leq\omega\leq2\omega_{0}$ (S78)

From equation S71, *α* ≠ -1, namely, *ω* cannot equal 0 according to equation S72. Namely,

$0<\omega\leq2\omega_{0}$ (S79)

Therefore for the n-layer FAMs, it will have *N* resonant frequencies, and they always between 0 and *ω*_0_/π. If each layer satisfies the impedance matching condition that *h*/*βd* = *c*_w_/*c*_a_, the resonant frequencies are also the frequencies of unity transmission. This conclusion has been confirmed with the FEM calculations, as shown in Fig. 3e-f.

**Note S7**

**The methods for trapping the air bubbles in water.**


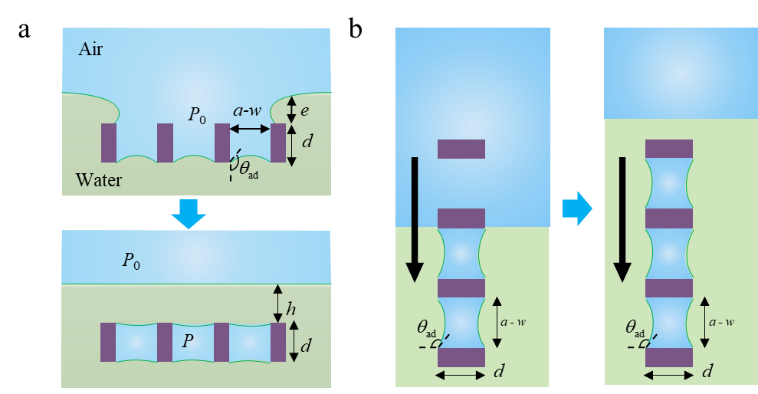


**Scheme S6**. (a) The horizontal immersion method when *d* is small. (b) The vertical immersion method for the case that *d* is large.

When a hydrophobic porous structure is immersed into water, the air might be trapped in the structure, namely, part of the structure cannot be wetted. This is called the Cassie state. Once the external pressure exceeds the critical pressure (*P*_break_) that breaks the Cassie state, the Cassie state will break down^6^. Therefore, for preparing the air layer successfully, the liquid static pressure must not be higher than *P*_break_. We have provided two methods for preparing the air layer, which is mainly determined by the thickness *d*. The horizontal immersion method in Scheme S6a is firstly considered. During the formation of the air layer, the bottom water-air interface will form many menisci that provide the Laplace pressure to resist the static pressure, forbidding the water penetrating into the cells. The maximum pressure^6,7^ that the menisci can provide equals *P*_break_, namely，

$P_{\text{break}}=-\frac{4\sigma\cos\theta_{\text{ad}}}{a-w}$ (S80)

where *σ* is the surface tension of water, *θ*_ad_ is the advancing angle on the solid surface. Equation S80 is a simplified equation by treating the cuboid solid cell as a cylinder with the bottom diameter of *a*-*w*. The maximum liquid static pressure must not be more than *P*_break_ before the upper menisci close, namely,

$P_{\text{break}}>\rho_{\text{w}}(d+e)g$ (S81)

where *g* is the acceleration of gravity, the *e* is the thickness of the water above the solid structure due to the hydrophobic effect and gravity. It can be calculated as following^8,9^.

$e=2\sqrt{\frac{\sigma}{\rho_{\text{w}}g}}\sin\frac{\theta_{\text{ad}}}{2}$ (S82)

Combing equation S80-S82, and introducing the capillary length that $k^{-1}=\sqrt{\sigma/\rho_{\text{w}}g}$, we can get

$(2k^{-1}\sin\frac{\theta_{\text{ad}}}{2}+d)(a-w)<-4k^{-2}\cos\theta_{\text{ad}}$ (S83)

Namely, when *d* is small and satisfies the above equation, the method in Scheme S6a can be used to prepare the air layer in water.

However, if the maximum transmission frequency is very low (50 Hz, for example), the *d* will be large and satisfying equation S83 is difficult. The vertical immersion method is provided for this case (Scheme S6b). The air bubbles are formed in the solid cells one by one, when all the bubbles in cells are formed, the sample can turn into the horizontal direction for the acoustic transmission.

A similar analysis is applied, namely, before the menisci close in each cell,

$P_{\text{break}}=-\frac{2\sigma}{(a-w)/2\cos\theta_{\text{ad}}}>\rho_{\text{w}}\frac{(a-w)}{2}g$ (S84)

The two menisci must not be touched before the menisci close, namely

$\frac{a-w}{2}<\frac{d}{2}$ (S85)

The above equation is obviously satisfied because *d* is large and the *a*-*w* is less than the capillary length (about 2.7 mm). From equation S84, the following equation can be obtained

$\frac{a-w}{\sqrt{-\cos\theta_{\text{ad}}}}<\sqrt{\frac{8\sigma}{\rho_{\text{w}}g}}$ (S86)

Substituting the *k* ^-1^ into the equation above,

$a-w<\sqrt{-8k^{-1}\cos\theta_{\text{ad}}}$ (S87)

Namely, when the condition in equation S87 are satisfied, the vertical immersion method can be used to prepare the air layer underwater.

**Note S8**

**The stability analysis for the prepared bubbles.**


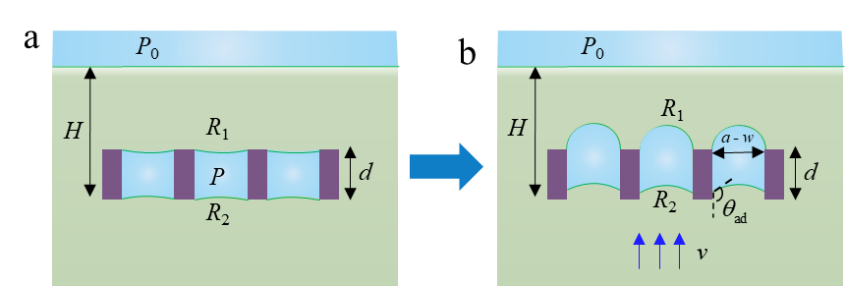


**Scheme S7**. (a) The formed bubbles in the cells. (b) The critical state that bubbles will depart from the cell.

Once the meniscus in Scheme S6a closed, bubbles will form into the cells. The bubble has two free air-water boundaries with the curvature radius of *R*_1_ and *R*_2_ (Scheme S7a). The pressure (*P*) in the bubbles will be balanced by the static pressure, atmospheric pressure (*P*_0_), and the Laplace pressure. For the bottom boundary,

$P+\frac{2\sigma}{R_{2}}=P_{0}+\rho_{\text{w}}Hg$ (S88)

Assuming the curvature of the bottom boundary (concave to the water) is positive, if the above boundary has the same direction (convex to the water), we can get

$P-\frac{2\sigma}{R_{1}}=P_{0}+\rho_{\text{w}}(H-d)g$ (S89)

From the two equations above,

$2\sigma(\frac{1}{R_{1}}+\frac{1}{R_{2}})=\rho_{\text{w}}dg$ (S90)

The equation above suggests, the sum of the curvature will not change with the immersion depth. Therefore, once the air-water interface is closed to form the bubbles, the stability of bubbles is not sensitive with the immersion depth. Actually, the processes in Scheme S6a is isobaric where the pressure in the solid cell is always *P*_0_, the static pressure must not be more than the breakdown pressure. Once the bubbles have been formed, the immersion process is isothermal. The pressure in the solid cell can increase by decreasing the bubble volume to resist the static pressure.

Next, we will demonstrate the bubbles can sustain a fluid speed of *v* that vertically acting on the bottom interface. The fluid speed has an additional pressure on the interface, namely, the dynamic pressure in the Bernoulli equation^10^, the equation S88 changes to

$P+\frac{2\sigma}{R_{2}}=P_{0}+\rho_{\text{w}}Hg+\frac{1}{2}\rho_{\text{w}}v^{2}$ (S91)

Assuming the fluid flow has little effect on the upper boundary, the equation of S89 doesn’t change, and equation S90 changes to

$2\sigma(\frac{1}{R_{1}}+\frac{1}{R_{2}})=\rho_{\text{w}}gd+\frac{1}{2}\rho_{\text{w}}v^{2}$ (S92)

If the speed is enough large, the bottom boundary will move once the contact angle is larger than the advancing angle *θ*_ad_. The critical state for the up boundary is the curvature radius equals (*a*-*w*)/2. After that, the boundary will move out of the cell. Therefore, at the critical state,

$R_{1}=\frac{a-w}{2\text{cos(}\pi-\theta_{\text{ad}})}$ $R_{2}=\frac{a-w}{2}$ (S93)

Substituting equation S93 into the equation S92, the allowed maximum velocity (*v*_max_) can be obtained

$v_{\max}=\sqrt{\frac{8\sigma(1-\cos\theta_{\text{ad}})}{\rho_{\text{w}}(a-w)}-2gd}$ (S94)

Substituting that *a* = 3 mm, *w* =1.2 mm, *d* = 5.1 mm, *θ*_ad_ = 135°, *σ =*72 mN/m, *g* = 9.8 N/Kg, *ρ*_w_ = 998 Kg/m^3^_,_ we can get that *v*_max_ = 0.67 m/s. Therefore, the bubbles can resist the vertical relative motion of the solid structure and water at a speed not more than 0.67 m/s. The horizontal motion was not limited because it has little influence on the air-water boundaries.

**Note S9**

**The temperature dependence of the acoustic metasurface.**

Three environmental factors could affect the stability of the acoustic metasurface above, namely, the external pressure, the air solubility in water and the temperature. Due to the air-layer is near the water surface, the air is almost saturated in the water, therefore the solubility effect can be negligible. When the air layer has been formed, at the temperature *T*_0_ and the immersion depth *h*, the ideal gas equation is

$(P_{0}+\rho_{\text{w}}hg)V_{0}=nRT_{0}$ (S95)

where *P*_0_ and *V*_0_ are the atmosphere pressure and volume, *n* is the amount of substance, and *R* is the ideal gas constant. The *h* is usually from millimeter to several centimeters, and the liquid static pressure is very small compared with the atmospheric pressure. Therefore, the effect of the pressure change on the FAM can also be negligible.


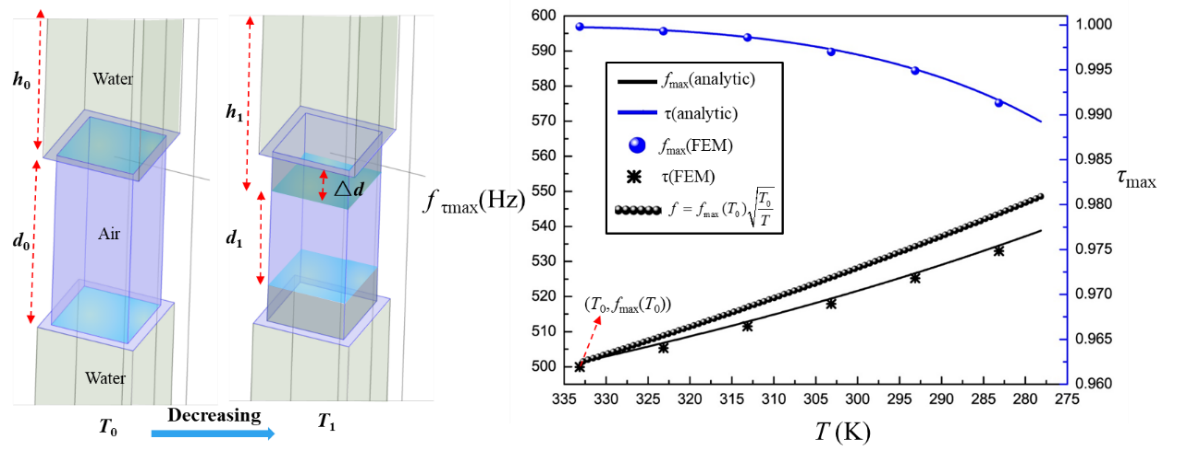


**Scheme S8.** The effect of temperature on the acoustic metasurface.

Next, we will discuss the effect of temperature on the maximum transmission frequency. Assuming that the system at a temperature *T*_0_, a decrease of temperature from *T*_0_ to *T*_1_ will reduce the thickness of *d*_0_ to *d*_1_. Half of the difference is marked with △*d*, as shown in Scheme S8.

$\Delta d=\frac{1}{2}(d_{1}-d_{0})$ (S96)

The decrease of air with be replaced by water, therefore the *h* will increase to *h*_1_. The difference is marked with △*h*. According to the ideal gas equation,

$P_{0}\Delta V=nR(T_{0}-T_{1})$ (S97)

where △*V* is decrease of air and it also equals the water that fills into the cell. Only half of △*V* contributes to change of *h*, namely,

$\Delta V=2S_{\text{d}}\Delta d=2S\Delta h$ (S98)

From the three equations above, *h*_1_ and *d*_1_ can be obtained

$$d_{1}\text{=}\frac{T_{1}}{T_{0}}d_{0}$$

$h_{1}=h_{0}+\frac{1}{2}(1-\frac{T_{1}}{T_{0}})d_{0}\beta$ (S99)

The acoustic speed, density of air and water will also be affected with temperature, they can be marked with *c*_a_(*T*), *ρ*_a_(*T*), *c*_w_(*T*) and *ρ*_w_(*T*). Substituting the relationship that $c_{\text{a}}=\sqrt{\gamma P_{0}/\rho_{\text{a}}}$ to the maximum transmission frequency that $\text{f}_{\text{max}}\text{= }\frac{c_{\text{a}}}{\text{2}\pi}\sqrt{\frac{\rho_{\text{a}}}{hd\beta\rho_{\text{w}}}}$, the maximum transmission frequency at different temperature *f*_max_(*T*) obeys

$f_{\text{max}}(T_{1})=\frac{\sqrt{\gamma P_{0}}}{2\pi\sqrt{h_{1}d_{1}\beta\rho_{\text{w}}(T_{1})}}=(\frac{1}{\sqrt{\frac{T_{1}\rho_{\text{w}}(T_{1})}{T_{0}\rho_{\text{w}}(T_{0})}[1+\frac{1}{2}(1-\frac{T_{1}}{T_{0}})\frac{c_{\text{a}}(T_{0})}{c_{\text{w}}(T_{0})}\text{]}}}-1)f_{\text{max}}(T_{0})=\alpha f_{\text{max}}(T_{0})$ (S100)

where

$\alpha\equiv(\frac{1}{\sqrt{\frac{T_{1}\rho_{w}(T_{1})}{T_{0}\rho_{w}(T_{0})}[1+\frac{1}{2}(1-\frac{T_{1}}{T_{0}})\frac{c_{a}(T_{0})}{c_{w}(T_{0})}\text{]}}}-1)$ (S101)

The relation of acoustic speed, density for air are

$c_{\text{a}}(T)=\sqrt{\frac{\gamma RT}{M}}$ $\rho_{\text{a}}(T)=\frac{P_{0}M}{RT}$ (S102)

where *γ*, *M* are adiabatic constant, the average relative molecular weight of air, respectively.

According to the material database in COMSOL MULTIPHYSICS 5.4, the acoustic speed, density for water for 5℃ to 90 ℃ approximatively follows below equations

$$\rho_{\text{w}}(T)=2.52875\times10^{-5}T^{3}-2.746\times10^{-2}T^{2}+9.3747T+27.76585$$

$c_{\text{w}}\text{(}T)\text{=2.49193}\times10^{\text{-4}}T^{3}-2.5847\times10^{\text{-1}}T^{2}+9.024312\times10^{\text{-1}}T-9038.62116$ (S103)

Given *T*_0_ = 335 K, the maximum transmission frequency is 500 Hz, substituting equation S102 and S103 into equation S100, the frequency with the maximum transmission coefficient varying with temperature can be plotted (Fig. 4h). It suggests, when temperature varies from 60 ℃ to 5 ℃, the super-transmission frequency will increase by about 10%. The energy transmission coefficient is also calculated with equation S58 by considering the temperature change, and the change is very small (from 100% to 99%). The FEM calculations agree with the analytical expression. If equation S102 and S103 are substituted into equation S101, we can get that

$\alpha\approx\sqrt{\frac{T_{0}}{T_{1}}}$ (S104)

Namely, when the temperature changes not too much, the maximum transmission frequency can be calculated approximately with the following equation

$f_{\text{max}}(T)\approx f_{\text{max}}(T_{0})\sqrt{\frac{T_{0}}{T}}$ (S105)

where *T* < *T*_0_.

**Note S10**

**The effect of the thermoviscous loss on the energy transmission coefficient**

The dissipation for the FAM mainly arises from the thermoviscous loss in the air layer near the resonant frequency^1^. The rigorous analysis of the loss can be performed by the fluid dynamics^11^. However, we can use the equivalent electrical transmission line model^12^ because the *d* is much less than the operating wavelength of sound. The air layer can be considered as a lumped element and the dissipation can be represented by an electrical resistance of$b_{\text{c}}/S_{\text{d}}^{2}$, where *b*_c_ is the real damping coefficient, *S*_d_ is the cross-sectional area of the air layer. The loss can be considered by adding an imaginary part of the mass of the water layer, namely, the effective mass (*m*_eff_) equals^1^

$m_{\text{eff}}=\rho_{\text{w}}hS-\frac{b_{\text{c}}}{\omega}i$ (S106)

Using the *m*_eff_ to replace the mass term of *ρ*_w_*Sh*, the energy transmission coefficient can be obtained as

$\tau=\frac{\frac{4Z_{\text{w}}}{Z_{\text{a}}}}{[(1+\frac{Z_{\text{w}}}{Z_{\text{a}}})\cos k_{\text{a}}d-\frac{\omega\rho_{\text{w}}hZ_{\text{w}}}{SZ_{\text{a}}^{2}}\beta\sin k_{\text{a}}d\text{+}\frac{b_{\text{c}}}{S^{2}Z_{\text{a}}}\cos k_{\text{a}}d]^{2}+[(\frac{1}{\beta}+\beta\frac{Z_{\text{w}}}{Z_{\text{a}}})\sin k_{\text{a}}d+\frac{(\omega\rho_{\text{w}}hS)}{S^{2}Z_{\text{a}}}\cos k_{\text{a}}d+\frac{b_{\text{c}}Z_{\text{w}}}{S^{2}Z_{\text{a}}^{2}}\beta\sin k_{\text{a}}d]^{2}}$ (S107)

The equation above changes to equation S58 when *b*_c_= 0. By analyzing the equation above and using the conditions that *k*_a_*d* << 1, sin*k*_a_*d* ≈ *k*_a_*d* and *Z*_w_ >> *Z*_a_, the maximum transmission also occurs at the following frequency,

$f_{\max}=\frac{c_{\text{a}}}{2\pi}\sqrt{(\frac{b_{\text{c}}}{S^{2}Z_{\text{w}}}+1)\frac{\rho_{\text{a}}}{\rho_{\text{w}}h\beta d}}$ (S108)

Because *b*_c_/*S*^2^*Z*_w_ is usually much less than 1, the *f*_max_ above is very close to the resonant frequency formula in Equation S59. Substituting the S108 into S107, the corresponding maximum energy transmission can be obtained as,

$\tau_{\max}=\frac{4}{(\frac{b_{\text{c}}}{S^{2}Z_{\text{w}}}+1)\text{[}\sqrt{x}(1+\frac{b_{\text{c}}}{S^{2}Z_{\text{a}}})+\sqrt{\frac{1}{x}}]^{2}}$ (S109)

where *x* = c_w_*βd*/c_a_*h*. The maximum value of *τ*_max_ occurs at

$\frac{1}{x}\text{=}(1+\frac{b_{\text{c}}}{S^{2}Z_{\text{a}}})$ (S110)

Namely, the *τ*_max_ satisfies,

$\tau_{\max}\leq\frac{1}{(1\text{+}\frac{b_{c}}{S^{2}Z_{w}})(1+\frac{b_{c}}{S^{2}Z_{a}})}<1$ (S111)

The equation above suggests, the existence of damping (*b*_c_ > 0) makes it impossible for 100% transmission of sound. Therefore the impedance matching condition has changed. It will exist the reflection and transmission waves. The *b*_c_ can be calculated with the FEM calculations. For the FAM in Fig.5b, *x* = *c*_w_*βd*/*c*_a_*h*= 0.9, and the transmission coefficient at 452 Hz is 0.17. Substituting them into equation S109, the *b*_c_ for the FAM at 452 Hz can be obtained as 0.011238 kg/s.

**Note S11**

**The impedance-matched FAM for the oblique incidence**


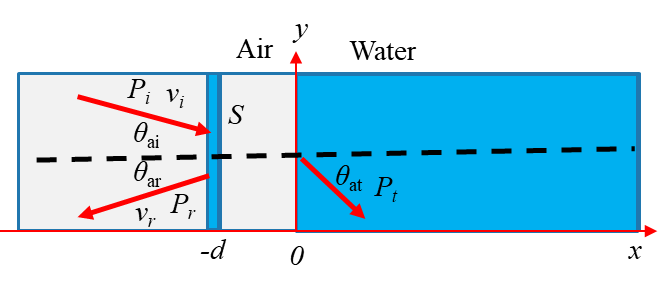


**Scheme S9.** The schematic diagram of the FAM for the oblique incidence case. The incident angle, reflection angle, refraction angle from air to water are *θ*_ai_, *θ*_ar_, *θ*_at_ respectively. Other parameters are the same with Scheme S2.

The acoustic metasurface in our system will obey the traditional Snell’s law^13,14^ . Namely, the refraction might occur at the air-water interface when the incident angle is not zero. As shown in Scheme S9, we only consider the case of from air to water acoustic transmission, because the case of water to air has the same transmission according to the reciprocity principle of acoustic wave equation^15,16^. Assuming the sound has an incident angle of *θ*_ai_ with the *x*-direction in the *xy*-plane, the refraction obeys the Snell’s law^17^

$\begin{matrix} \frac{c_{\text{a}}}{c_{\text{w}}}=\frac{\sin\theta_{\text{ai}}}{\sin\theta_{\text{at}}} \\ \theta_{\text{ai}}=\theta_{\text{ar}} \end{matrix}$ (S112)

For studying air-water transmission, *θ*_ai_ should be less than the critical angle of total reflection,

$\theta_{\text{ai}}<\arcsin\frac{c_{\text{a}}}{c_{\text{w}}}\approx13.4^{^{\circ}}$ (S113)

The *Z*_as_, *Z*_ws_ are the normal characteristic acoustic impedance of air and water, respectively.

$\begin{matrix} Z_{\text{as}}=\frac{\rho_{\text{a}}c_{\text{a}}}{S\cos\theta_{\text{ai}}} \\ Z_{\text{ws}}=\frac{\rho_{\text{w}}c_{\text{w}}}{S\cos\theta_{\text{at}}} \end{matrix}$ (S114)

The wave equation in Scheme S9 is $\varepsilon=\varepsilon_{0}e^{i(-k_{\text{a}}x\cos\theta_{\text{ai}}+k_{\text{a}}y\sin\theta_{\text{ai}})}$. The acoustic pressure (*P*_I_), particle velocity (*v*_I_) at *x* = -*d* are

$\begin{matrix} P_{\text{I}}=P_{\text{i}}+P_{\text{r}}=P_{\text{i,a}}e^{i(k_{\text{a}}d\cos\theta_{\text{ai}}+k_{\text{a}}y\sin\theta_{\text{ai}})}+P_{\text{r,a}}\text{e}^{i(-k_{\text{a}}d\cos\theta_{\text{ai}}+k_{\text{a}}y\sin\theta_{\text{ai}})} \\ v_{\text{I}}=\frac{P_{\text{i,a}}e^{i(k_{\text{a}}d\cos\theta_{\text{ai}}+k_{\text{a}}y\sin\theta_{\text{ai}})}}{SZ_{\text{as}}}-\frac{P_{\text{r,a}}\text{e}^{i(-k_{\text{a}}d\cos\theta_{\text{ai}}+k_{\text{a}}y\sin\theta_{\text{ai}})}}{SZ_{\text{as}}} \end{matrix}$ (S115)

The acoustic impendence (*Z*_ds_) at the *x* = -*d* changes to

$Z_{\text{ds}}=\frac{P_{\text{I}}}{\text{v}_{\text{I}}S}=\frac{P_{\text{i,a}}e^{i(k_{\text{a}}d\cos\theta_{\text{ai}}+k_{\text{a}}y\sin\theta_{\text{ai}})}+P_{\text{r,a}}\text{e}^{i(-k_{\text{a}}d\cos\theta_{\text{ai}}+k_{\text{a}}y\sin\theta_{\text{ai}})}}{\frac{P_{\text{i,a}}e^{i(k_{\text{a}}d\cos\theta_{\text{ai}}+k_{\text{a}}y\sin\theta_{\text{ai}})}}{SZ_{\text{as}}}-\frac{P_{\text{r,a}}\text{e}^{i(-k_{\text{a}}d\cos\theta_{\text{ai}}+k_{\text{a}}y\sin\theta_{\text{ai}})}}{SZ_{\text{as}}}}$ (S116)

Similar to equation S8, the reflection at *x* = 0 shows that

$\frac{P_{\text{r,a}}}{P_{\text{i,a}}}=\frac{Z_{\text{ws}}-Z_{\text{as}}}{Z_{\text{ws}}+Z_{\text{as}}}$ (S117)

Substituting equation S117 into S116 and simplifying,

$Z_{\text{ds}}=\frac{Z_{\text{ws}}}{1+(\frac{Z_{\text{ws}}^{2}}{Z_{\text{as}}^{2}}-1)\sin^{2}(k_{\text{a}}d\cos\theta_{\text{ai}})}-i\frac{Z_{\text{as}}}{2}\frac{(\frac{Z_{\text{ws}}^{2}}{Z_{\text{as}}^{2}}-1)\sin(2k_{\text{a}}d\cos\theta_{\text{ai}})}{1+(\frac{Z_{\text{ws}}^{2}}{Z_{\text{as}}^{2}}-1)\sin^{2}(k_{\text{a}}d\cos\theta_{\text{ai}})}$ (S118)

By inserting the mass term into equation S118, the acoustic impedance can be achieved.

$Z_{\text{ds}}=\frac{Z_{\text{ws}}}{1+(\frac{Z_{\text{ws}}^{2}}{Z_{\text{as}}^{2}}-1)\sin^{2}(k_{\text{a}}d\cos\theta_{\text{ai}})}+i(\frac{k_{\text{a}}h\rho_{\text{w}}c_{\text{a}}}{S}-\frac{Z_{\text{as}}}{2}\frac{(\frac{Z_{\text{ws}}^{2}}{Z_{\text{as}}^{2}}-1)\sin(2k_{\text{a}}d\cos\theta_{\text{ai}})}{1+(\frac{Z_{\text{ws}}^{2}}{Z_{\text{as}}^{2}}-1)\sin^{2}(k_{\text{a}}d\cos\theta_{\text{ai}})})$ (S119)

The reflection coefficient (*R*_ds_) at the position *x* = *-d* is

$R_{\text{ds}}=\frac{Z_{\text{ds}}-Z_{\text{as}}}{Z_{\text{ds}}+Z_{\text{as}}}$ (S120)

Therefore the IMC changes to Z_ds_ = Z_as_, and the following equations will be achieved:

$\begin{matrix} \frac{Z_{\text{ws}}}{1+(\frac{Z_{\text{ws}}^{2}}{Z_{\text{as}}^{2}}-1)\sin^{2}(k_{\text{a}}d\cos\theta_{\text{ai}})}=Z_{\text{as}} \\ \frac{k_{\text{a}}h\rho_{\text{w}}c_{\text{a}}}{S}-\frac{Z_{\text{as}}}{2}\frac{(\frac{Z_{\text{ws}}^{2}}{Z_{\text{as}}^{2}}-1)\sin(2k_{\text{a}}d\cos\theta_{\text{ai}})}{1+(\frac{Z_{\text{ws}}^{2}}{Z_{\text{as}}^{2}}-1)\sin^{2}(k_{\text{a}}d\cos\theta_{\text{ai}})}=0 \end{matrix}$ (S121)

Substituting the conditions that *k*_a_*d* << 1, sin*k*_a_*d* ≈ *k*_a_*d*, Z_as_ << Z_ws_ and equation S114 to equation S121, the following equations can be achieved

$d=\sqrt{\frac{\rho_{\text{a}}c_{\text{a}}}{\rho_{\text{w}}c_{\text{w}}}\frac{\cos\theta_{\text{at}}}{\cos\theta_{\text{ai}}}}\frac{1}{k_{\text{a}}\cos\theta_{\text{ai}}}$ $h=\sqrt{\frac{c_{\text{w}}\rho_{\text{a}}}{c_{\text{a}}\rho_{\text{w}}}\frac{1}{\cos\theta_{\text{at}}\cos\theta_{\text{ai}}}}\frac{1}{k_{\text{a}}}$

$\frac{h}{d}=\frac{c_{\text{w}}\cos\theta_{\text{ai}}}{c_{\text{a}}\cos\theta_{\text{at}}}$ $f_{\text{us}}=\frac{c_{\text{a}}}{2\pi\cos\theta_{\text{ai}}}\sqrt{\frac{\rho_{\text{a}}}{\rho_{\text{w}}}\frac{1}{hd}}$ (S122)

where *f*_us_ is the frequency of unity transmission for the air-to-water oblique incidence. Comparison equation S122 with equation S15-S18 suggests that the frequency of unity transmission slightly increases with the incident angle, namely,

$f_{\text{us}}=\frac{f_{\text{u}}}{\cos\theta_{\text{ai}}}\text{=}\frac{f_{\text{u}}}{\sqrt{1-\frac{c_{\text{a}}^{2}}{c_{\text{w}}^{2}}\sin^{2}\theta_{\text{wi}}}}$ (S123)

where *θ*_wi_ is the incident form water to air. According to equation S113, the allowed incident angle of *θ*_ai_ is very small (from 0 to 13.4°), therefore the change in frequency of *f*_us_ with *θ*_ai_ is also very small. It is also suggested that if a sound wave transmits from water into air, the changes of the frequency will be less than 3% even the incident angle (*θ*_wi_) changes from 0 to 90°.

If the *θ*_wi_ is large, the IMC changes a lot. The *h*/*d* = *c*_w_/*c*_a_ is only suitable for the normal incidence. With the increasing incidence angle, the energy transmission coefficient will not be equal to 1. By considering the parameter of *β*, the IMC changes to

$\frac{h}{\beta d}=\frac{c_{\text{w}}\cos\theta_{\text{ai}}}{c_{\text{a}}\cos\theta_{\text{at}}}$ (S124)

**Note S12**

**Calculating the transmission coefficients for the oblique incidence**


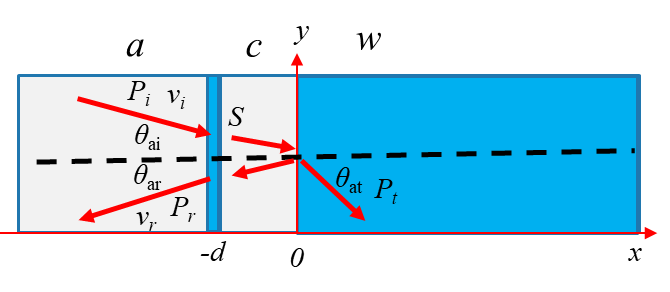


**Scheme S10.** The schematic diagram of the FAM for the oblique incidence. All parameters are the same with Scheme S9. The air layer in the water divides the space into three parts of *a*, *c* and *w*.

Assuming the acoustic wave has an incident angle of *θ*_ai_ with the *x*-direction in the *xy*-plane (Scheme S10), the water layer is also believed to oscillate integrally, and the sound propagating within the water layer is not considered. The sound wave doesn’t change the direction while across the water layer because the thickness is much less than the wavelength. The acoustic pressure and particle velocity at different positions are marked with *P*(*x*) and *v*(*x*), respectively.

For *x* < -*d*,

$\begin{matrix} P^{\text{a}}(x)=P_{\text{i,a}}^{\text{a}}e^{i(-k_{\text{a}}x\cos\theta_{\text{ai}}+k_{\text{a}}y\sin\theta_{\text{ai}})}+P_{\text{r,a}}^{\text{a}}e^{i(k_{\text{a}}x\cos\theta_{\text{ai}}+k_{\text{a}}y\sin\theta_{\text{ai}})} \\ u^{\text{a}}(x)=\frac{P_{\text{i,a}}^{\text{a}}e^{i(-k_{\text{a}}x\cos\theta_{\text{ai}}+k_{\text{a}}y\sin\theta_{\text{ai}})}}{SZ_{\text{as}}}-\frac{P_{\text{r,a}}^{\text{a}}e^{i(k_{\text{a}}x\cos\theta_{\text{ai}}+k_{\text{a}}y\sin\theta_{\text{ai}})}}{SZ_{\text{as}}} \end{matrix}$ (S125)

For – *d* < *x* < 0,

$\begin{matrix} P^{\text{c}}(x)=P_{\text{i,a}}^{\text{c}}e^{i(-k_{\text{a}}x\cos\theta_{\text{ai}}+k_{\text{a}}y\sin\theta_{\text{ai}})}+P_{\text{r,a}}^{\text{c}}e^{i(k_{\text{a}}x\cos\theta_{\text{ai}}+k_{\text{a}}y\sin\theta_{\text{ai}})} \\ u^{\text{c}}(x)=\frac{P_{\text{i,a}}^{\text{c}}e^{i(-k_{\text{a}}x\cos\theta_{\text{ai}}+k_{\text{a}}y\sin\theta_{\text{ai}})}}{SZ_{\text{as}}}-\frac{P_{\text{r,a}}^{\text{c}}e^{i(k_{\text{a}}x\cos\theta_{\text{ai}}+k_{\text{a}}y\sin\theta_{\text{ai}})}}{SZ_{\text{as}}} \end{matrix}$ (S126)

For 0 < *x*,

$\begin{matrix} P^{\text{w}}(x)=P_{\text{t,a}}^{\text{w}}e^{i(-k_{\text{w}}x\cos\theta_{\text{at}}+k_{\text{w}}y\sin\theta_{\text{at}})} \\ u^{\text{w}}(x)=\frac{P_{\text{t,a}}^{\text{w}}e^{i(-k_{\text{w}}x\cos\theta_{\text{at}}+k_{\text{w}}y\sin\theta_{\text{at}})}}{SZ_{\text{ws}}} \end{matrix}$ (S127)

The total transmission coefficient *T*_s_ is

$T_{\text{s}}=\frac{\left.P_{\text{t,a}}^{\text{w}}e^{i(-k_{\text{w}}x\cos\theta_{\text{at}}+k_{\text{w}}y\sin\theta_{\text{at}})}\right|_{x=0}}{\left.P_{\text{i,a}}^{\text{a}}e^{i(-k_{\text{a}}x\cos\theta_{\text{ai}}+k_{\text{a}}y\sin\theta_{\text{ai}})}\right|_{x=-d}}=\frac{P_{\text{t,a}}^{\text{w}}e^{i(k_{\text{w}}y\sin\theta_{\text{at}})}}{P_{\text{i,a}}^{\text{a}}e^{i(k_{\text{a}}\text{d}\cos\theta_{\text{ai}}+k_{\text{a}}y\sin\theta_{\text{ai}})}}$ (S128)

The reflection coefficient *R*_s_ is

$R_{\text{s}}=\frac{\left.P_{\text{r,a}}^{\text{a}}e^{i(k_{\text{a}}x\cos\theta_{\text{ai}}+k_{\text{a}}y\sin\theta_{\text{ai}})}\right|_{x=-d}}{\left.P_{\text{i,a}}^{\text{a}}e^{i(-k_{\text{a}}x\cos\theta_{\text{ai}}+k_{\text{a}}y\sin\theta_{\text{ai}})}\right|_{x=-d}}=\frac{P_{\text{r,a}}^{\text{a}}}{P_{\text{i,a}}^{\text{a}}e^{i2k_{\text{a}}d\cos\theta_{\text{ai}}}}$ (S129)

The reflection coefficient *R*_cs_ at *x* = 0 is

$R_{\text{cs}}=\frac{\left.P_{\text{r,a}}^{\text{c}}e^{i(k_{\text{a}}x\cos\theta_{\text{ai}}+k_{\text{a}}y\sin\theta_{\text{ai}})}\right|_{x=0}}{\left.P_{\text{i,a}}^{\text{c}}e^{i(-k_{\text{a}}x\cos\theta_{\text{ai}}+k_{\text{a}}y\sin\theta_{\text{ai}})}\right|_{x=0}}=\frac{P_{\text{r,a}}^{\text{c}}}{P_{\text{i,a}}^{\text{c}}}$ (S130)

The transmission coefficient *T*_cs_ at *x* = -*d* is

$T_{\text{cs}}=\frac{\left.P_{\text{i,a}}^{\text{c}}e^{i(-k_{\text{a}}x\cos\theta_{\text{ai}}+k_{\text{a}}y\sin\theta_{\text{ai}})}\right|_{x=-d}}{\left.P_{\text{i,a}}^{\text{a}}e^{i(-k_{\text{a}}x\cos\theta_{\text{ai}}+k_{\text{a}}y\sin\theta_{\text{ai}})}\right|_{x=-d}}=\frac{P_{\text{i,a}}^{\text{c}}}{P_{\text{i,a}}^{\text{a}}}$ (S131)

The boundary conditions at *x* = -*d* are:

$$(\left.P^{\text{a}}\right|_{x=-d}\left.-P^{\text{c}}\right|_{x=-d})S=m_{\text{c}}\frac{\left.du^{\text{c}}\right|_{x=-d}}{dt}=i\omega m_{\text{c}}\left.u^{\text{c}}\right|_{x=-d}$$

$\left.u^{\text{a}}\right|_{x=-d}=\left.u^{\text{c}}\right|_{x=-d}$ (S132)

where *m*_c_ is the mass of the water, and equals *ρ*_w_*hS*_._ The boundary conditions at *x* = 0 are:

$$\left.P^{\text{c}}\text{(}x\text{)}\right|_{x=0}=\left.P^{\text{w}}\text{(}x\text{)}\right|_{x=0}$$

$\left.u^{\text{c}}\right|_{x=0}=\left.u^{\text{w}}\right|_{x=0}$ (S133)

Substituting equation S125, S129, S131 into equation S132, we can get

$$1+R_{\text{s}}=T_{\text{cs}}[\frac{iw\rho_{w}h}{Z_{\text{as}}}(1-R_{\text{cs}}e^{i(-2k_{\text{a}}d\cos\theta_{\text{ai}})})+1+R_{\text{cs}}e^{i(-2k_{\text{a}}d\cos\theta_{\text{ai}})}]$$

$1-R_{\text{s}}=T_{\text{cs}}(1-R_{\text{cs}}e^{i(-2k_{\text{a}}d\cos\theta_{\text{ai}})})$ (S134)

Then from equation S128, the *T*_cs_ can be obtained

$T_{\text{cs}}=\frac{2}{2+iw\frac{\rho_{\text{w}}h}{Z_{\text{as}}}(1-R_{\text{cs}}e^{-i2k_{\text{a}}d\cos\theta_{\text{ai}}})}$ (S135)

Substituting equation S130, S131, S126-S128 into equation S133,

$$1+R_{\text{cs}}=\frac{T_{\text{s}}}{T_{\text{cs}}}e^{ik_{\text{a}}d\cos\theta_{\text{ai}}}$$

$1-R_{\text{cs}}=\frac{Z_{\text{as}}}{Z_{\text{ws}}}\frac{T}{T_{\text{cs}}}e^{ik_{\text{a}}d\cos\theta_{\text{ai}}}$ (S136)

From equation S136, we can get

$$R_{\text{cs}}=\frac{Z_{\text{ws}}-Z_{\text{as}}}{Z_{\text{ws}}+Z_{\text{as}}}$$

$T_{\text{s}}=2T_{\text{cs}}e^{-ik_{\text{a}}d\cos\theta_{\text{ai}}}\frac{Z_{\text{ws}}}{Z_{\text{ws}}+Z_{\text{as}}}$ (S137)

From equation S137 and S135, the total transmission coefficient *T*_s_ can be obtained

$T_{\text{s}}=\frac{\frac{2Z_{\text{ws}}}{Z_{\text{as}}}}{(1+\frac{Z_{\text{ws}}}{Z_{\text{as}}})\cos(k_{\text{a}}d\cos\theta_{\text{ai}})-\frac{\omega\rho_{\text{w}}hZ_{\text{ws}}}{SZ_{\text{as}}^{2}}\sin(k_{\text{a}}d\cos\theta_{\text{ai}})+i[(1+\frac{Z_{\text{ws}}}{Z_{\text{as}}})\sin k_{\text{a}}d\cos\theta_{\text{ai}}+\frac{\omega\rho_{\text{w}}h}{SZ_{\text{as}}}\cos(k_{\text{a}}d\cos\theta_{\text{ai}})]}$ (S138)

The energy transmission coefficient (*τ*_s_) can be also achieved

$\tau_{\text{s}}=\frac{\frac{4Z_{\text{ws}}}{Z_{\text{as}}}}{[(1+\frac{Z_{\text{ws}}}{Z_{\text{as}}})\cos(k_{\text{a}}d\cos\theta_{\text{ai}})-\frac{\omega\rho_{\text{w}}hZ_{\text{ws}}}{SZ_{\text{as}}^{2}}\sin(k_{\text{a}}d\cos\theta_{\text{ai}})]^{2}+[(1+\frac{Z_{\text{ws}}}{Z_{\text{as}}})\sin(k_{\text{a}}d\cos\theta_{\text{ai}})+\frac{\omega\rho_{\text{w}}h}{SZ_{\text{as}}}\cos(k_{\text{a}}d\cos\theta_{\text{ai}})]^{2}}$ (S139)

The equation above gives the energy transmission coefficient for oblique incidence from air to water. If the oblique incidence is from water to air with the incident angle of *θ*_wi_, it will exactly equal the refraction angle of *θ*_at_ from air to water. Similarly, the refraction angle from water to air (*θ*_wt_) equals the incident angle from air to water (*θ*_ai_). From equation S112, we can get that

$\cos\theta_{\text{ai}}=\sqrt{1-\frac{c_{\text{a}}^{2}}{c_{\text{w}}^{2}}\sin^{2}\theta_{\text{at}}}=\sqrt{1-\frac{c_{\text{a}}^{2}}{c_{\text{w}}^{2}}\sin^{2}\theta_{\text{wi}}}$ (S140)

Substituting equation S140 into S139, the energy transmission coefficient from water to air with an incident angle of *θ*_wi_ is

$\tau_{\text{s}}=\frac{\frac{4Z_{\text{ws}}}{Z_{\text{as}}}}{[(1+\frac{Z_{\text{ws}}}{Z_{\text{as}}})\cos(k_{\text{a}}d\sqrt{1-\frac{c_{\text{a}}^{2}}{c_{\text{w}}^{2}}\sin^{2}\theta_{\text{wi}}})-\frac{\omega\rho_{\text{w}}hZ_{\text{ws}}}{SZ_{\text{as}}^{2}}\sin(k_{\text{a}}d\sqrt{1-\frac{c_{\text{a}}^{2}}{c_{\text{w}}^{2}}\sin^{2}\theta_{\text{wi}}})]^{2}+[(1+\frac{Z_{\text{ws}}}{Z_{\text{as}}})\sin(k_{\text{a}}d\sqrt{1-\frac{c_{\text{a}}^{2}}{c_{\text{w}}^{2}}\sin^{2}\theta_{\text{wi}}})+\frac{\omega\rho_{\text{w}}h}{SZ_{\text{as}}}\cos(k_{\text{a}}d\sqrt{1-\frac{c_{\text{a}}^{2}}{c_{\text{w}}^{2}}\sin^{2}\theta_{\text{wi}}})]^{2}}$ (S141)

where $Z_{\text{as}}=\frac{\rho_{\text{a}}c_{\text{a}}}{S\cos\theta_{\text{ai}}}=\frac{\rho_{\text{a}}c_{\text{a}}}{S\cos\theta_{\text{wt}}}$ and $Z_{\text{ws}}=\frac{\rho_{\text{w}}c_{\text{w}}}{S\cos\theta_{\text{at}}}=\frac{\rho_{\text{w}}c_{\text{w}}}{S\cos\theta_{\text{wi}}}$.

The equation S141 gives the energy transmission coefficient for any incident angle from water to air, which agrees well with the FEM and IMC calculations (Fig. S10).


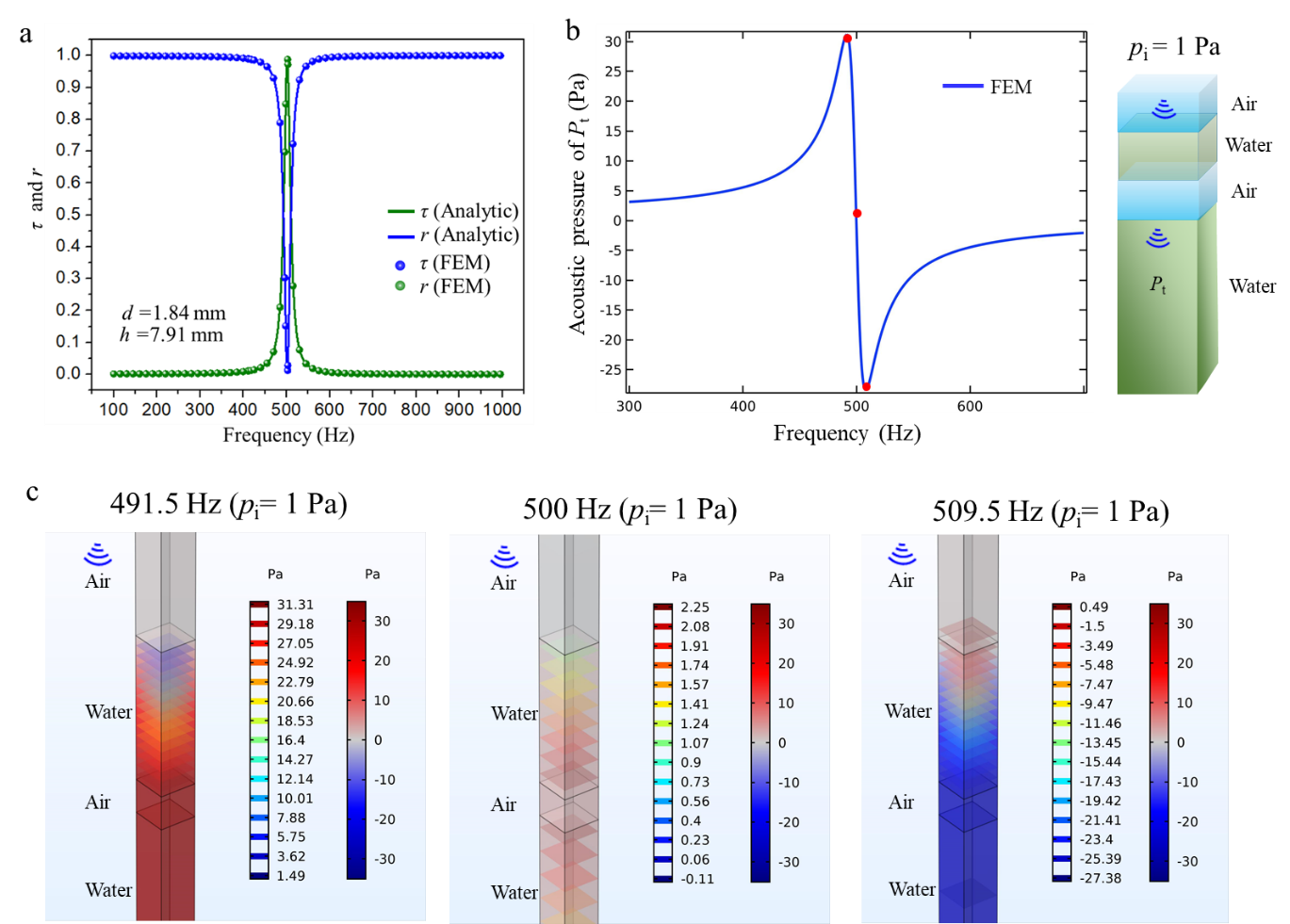


**Fig. S1. (a)** The analytical solution in Note S3 agrees well with the FEM and the IMC calculations. The parameters are *h* = 7.91 mm, *d* = 1.84 mm, *c*_a_ = 344 m/s, *c*_w_ = 1480 m/s, *ρ*_a_ = 1.21 Kg/m^3^, and *ρ*_w_ = 998 Kg/m^3^. The same sound speed and density of air and water are also used in FEM and other calculations. As shown above, the energy transmission coefficient (*τ*) and the energy reflection coefficient (*r*) in the analytical solution agree with the FEM calculations. Besides, the IMC also predicts the frequency of unity transmission at 499.7 Hz. (**b**) The acoustic pressure of the transmitted wave in water varies with frequency. The incident wave has a pressure of 1 Pa. The sampling point is near the second water-air interface. The red points are the frequencies shown in **c**. (**c**) The field patterns of acoustic pressure at 491.5 Hz, 500 Hz, and 509.5 Hz. They show that near the resonant frequency, the acoustic pressure of the transmitted wave is magnified by over 30 Pa, and the amplitude is over 60 Pa. The viscous and thermal losses are not considered.


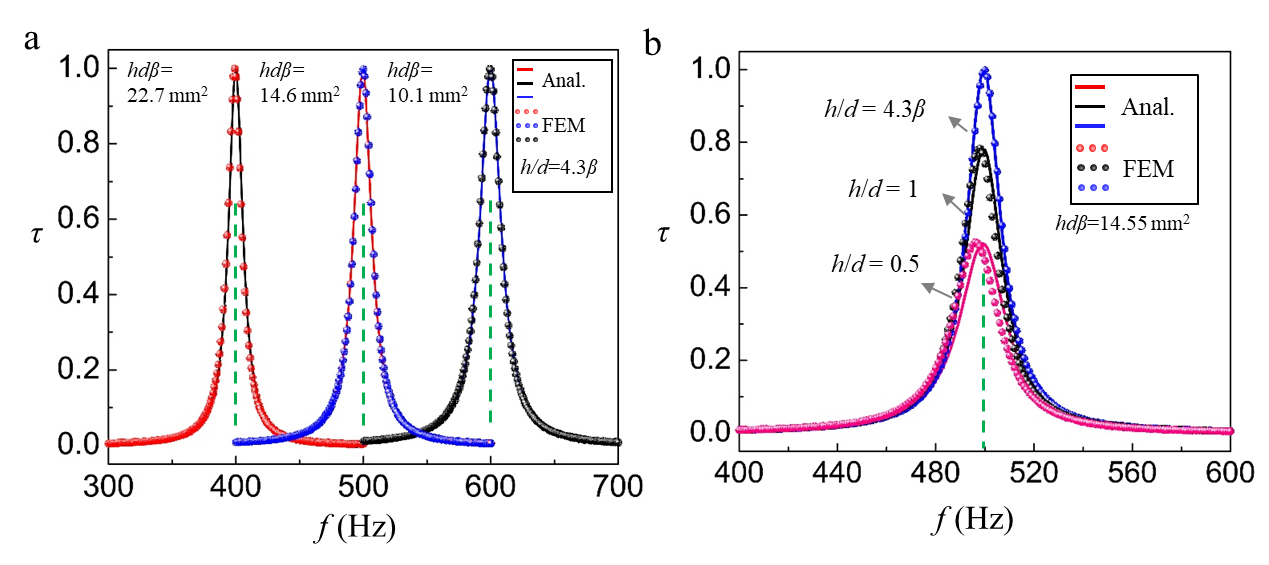


**Fig. S2. The analytical solution in Note S5 agrees well with the FEM and the impedance matching condition calculation after considering the effect of solid structure.** (**a**) The maximum transmission frequency can be adjusted by changing the value of *hdβ* and keeping that *h*/*d*=*βc*_w_/*c*_a_=4.3*β*. The maximum energy transmission coefficient also reaches 1 because the IMC are satisfied. (**b**) The maximum energy transmission can be adjusted by changing the value of *h*/*d*. The frequency at the maximum of *τ* doesn’t change because the *hdβ* is kept at 14.55 mm^2^. In the FEM calculations above, *a* =1.5 mm and *w* = 0.3 mm, namely, *β*= 0.64. The *h* and *d* are changeable for adjusting the frequency of unity transmission and the maximum of *τ*. The viscous and thermal losses are not considered.


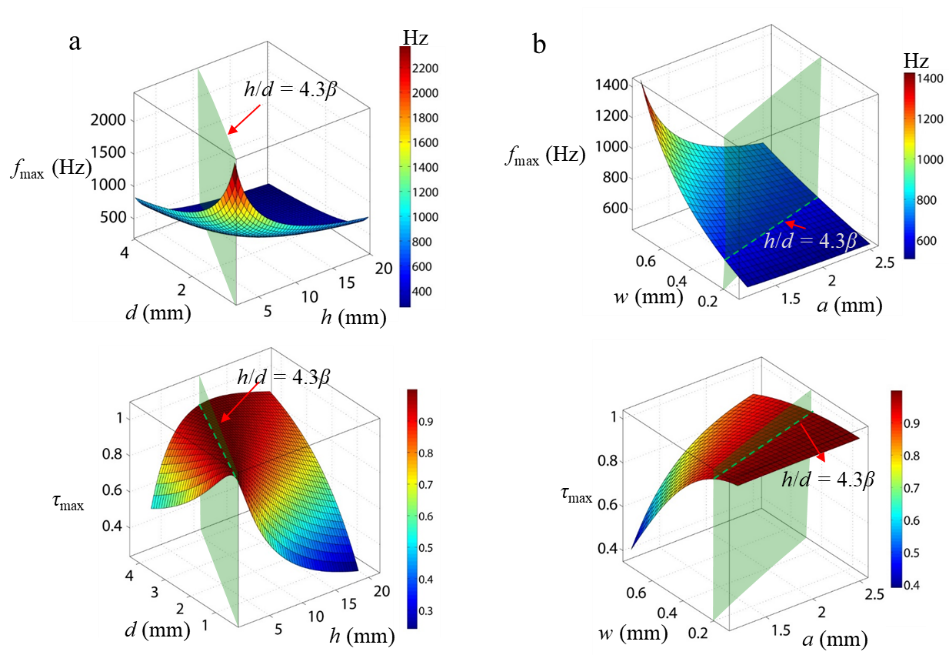


**Fig. S3.** The effects of *h*, *d*, *a* and *w* on the maximum transmission frequency of *f*_max_ and the corresponding energy transmission coefficient *τ*_max_. (**a**) The effects of *h* and *d* by keeping *a* =1.5 mm, *w* = 0.3 mm, namely, *β*= 0.64. (**b**) The effect of *a* and *w* by keeping that *h* = 7.91 mm, *d* = 1.84 mm. The IMC that *h*/*d*=4.3*β* where *τ*_max_ = 1 is indicated.


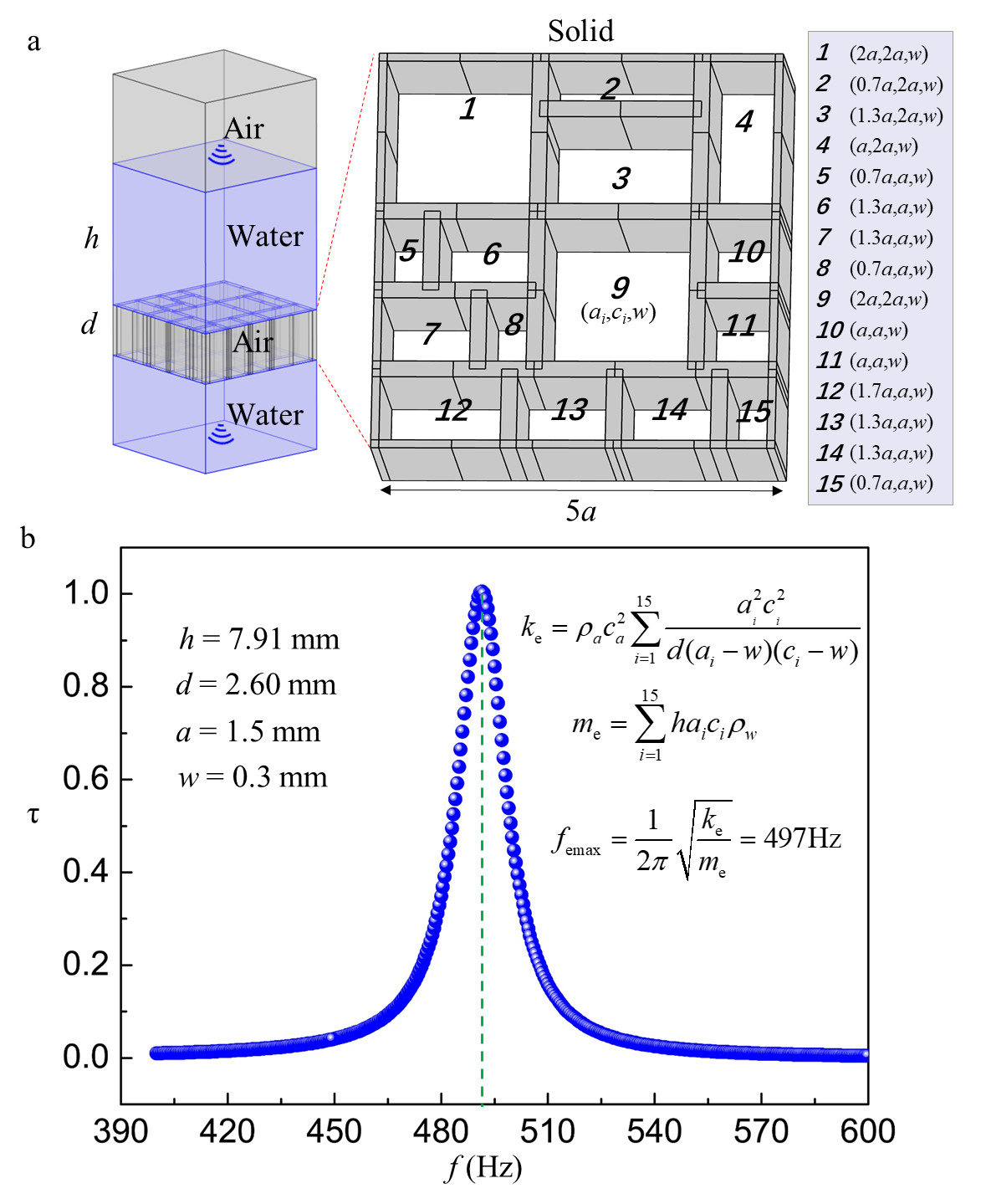


**Fig. S4. Bubbles with different parameters can be treated as the parallel mass-spring model.** (**a**) The model used for the FEM calculation and the parameters in each cell. (**b**) The analytical solution (*f*_emax_ = 497 Hz) predicts the maximum transmission frequency that agrees well with the FEM calculation (blue dots). The viscous and thermal losses above are not considered.


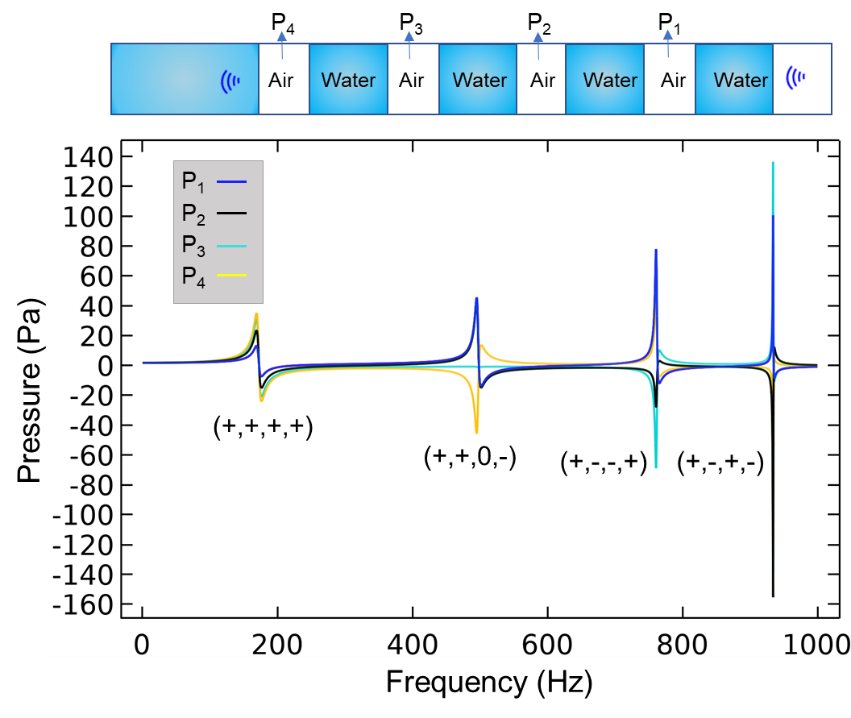


**Fig. S5.** The modes of vibration at different frequencies of unity transmission for *n* = 4. Given that the air compression is positive, the positive pressure in the air layer is marked with “+”. The pressure given here is the relative pressure normalized by the atmospheric pressure. The viscous and thermal losses are not considered.


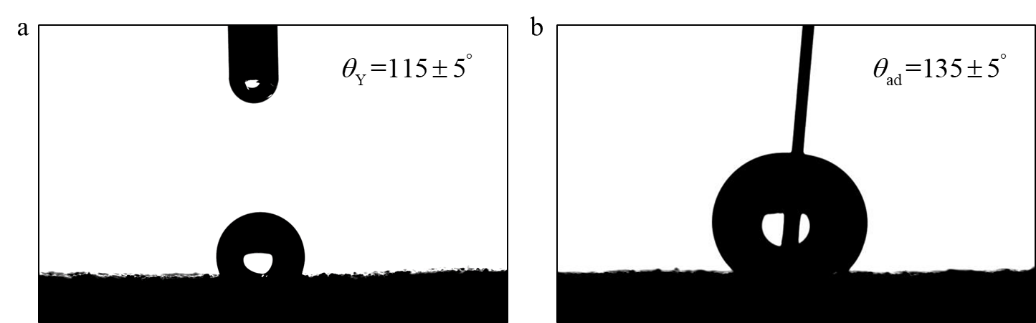


**Fig. S6.** The contact angle *θ*_Y_ (**a**), and the advancing angle *θ*_ad_ (**b**) on the surface of the printed nylon solid structure.


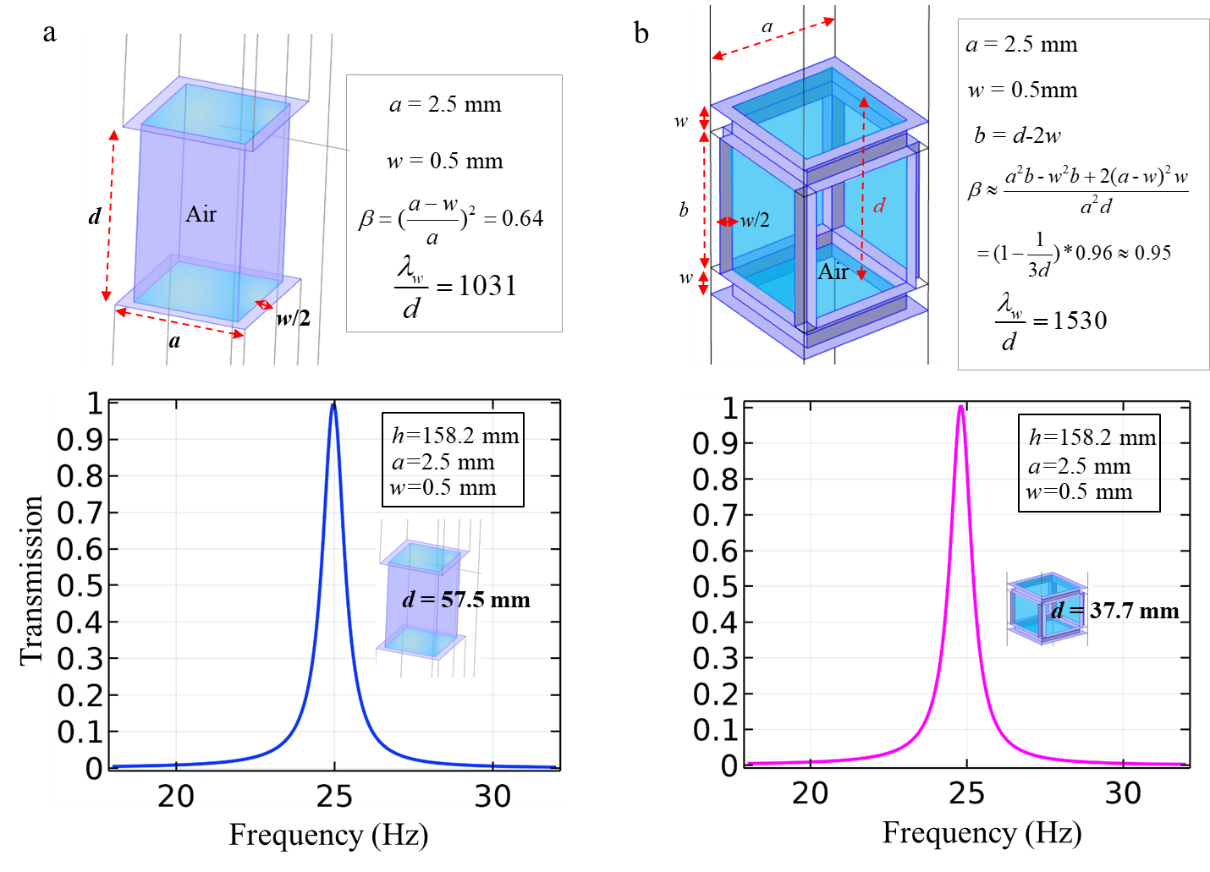


**Fig. S7. The ultrathin property of the FAM.** (**a**) The wall structure has a ratio of wavelength-to-thickness ratio *λ*_w_/*d* = 1031, where *λ*_w_ is the wavelength in water and *d* is the thickness of the solid structure. (**b**) The ratio can be even larger with a frame structure. For example, the FEM calculations above suggest that for the frequency of unity transmission at 25 Hz, the wall structure needs a thickness of 57.5 mm, but the frame structure only requires a thickness of 37.7 mm. The viscous and thermal losses are not considered.


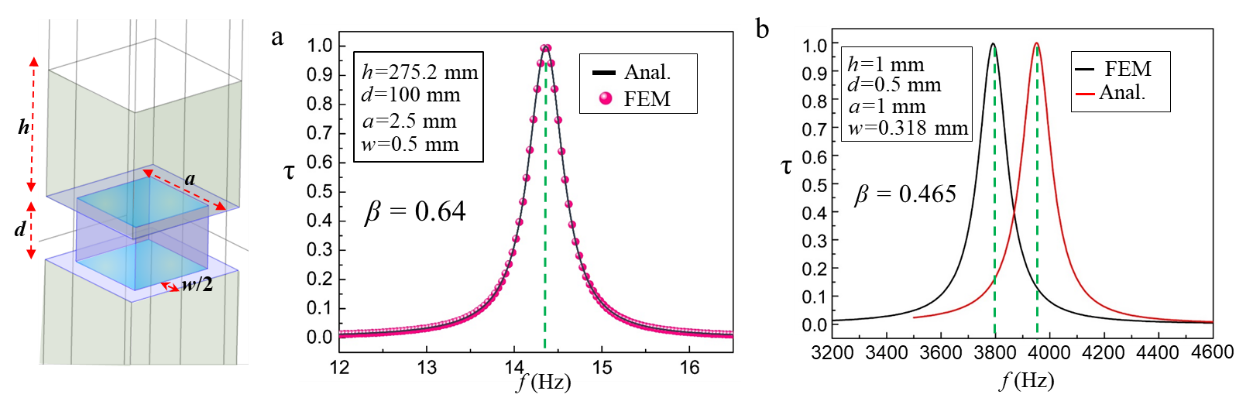


**Fig. S8. The operating frequency range for the FAM.** (**a**) Assuming that the common 3D printing thickness is limited to 10 cm, the lowest frequency that satisfying the IMC is about 14 Hz. (**b**) Assuming that the water layer less than 1 mm is impractical to prepare, the maximum frequency of unity transmission that satisfying the IMC is about 4000 Hz. The difference between the analytical solution and FEM results arises from the solid confinement effect on the water layer when *β* is small. The viscous and thermal losses are not considered.

**
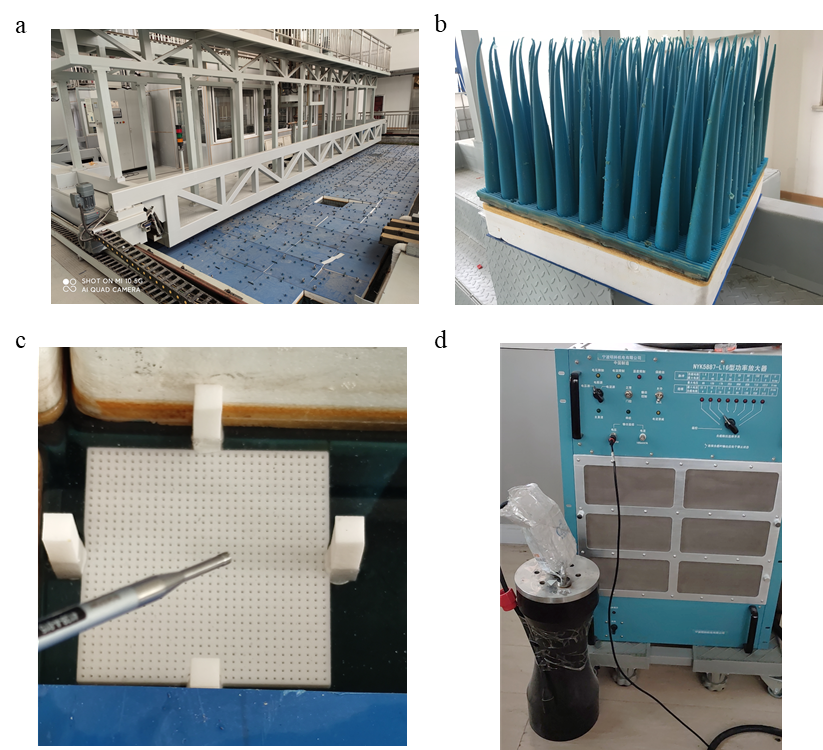
**

**Fig. S9. The acoustic experiments for demonstrating the acoustic performance of the FAM.** (**a**) The water pool with the size of 20 m ×12 m ×8 m, surrounding by the absorbing wedges. (**b**) The absorbing wedges. The water pool and the surrounding absorbing wedge are not effective enough for absorbing the sound at near 450 Hz for preventing reflections. Here the setup is mainly used to measure the frequency of the maximum transmission, and the transmission with and without the FAM can be used to qualitatively exhibit the transmission enhancement. (**c**) The receiving probe and the FAM, surrounding by the absorbing wedges. (**d**) The emission sound source and the power amplifier.


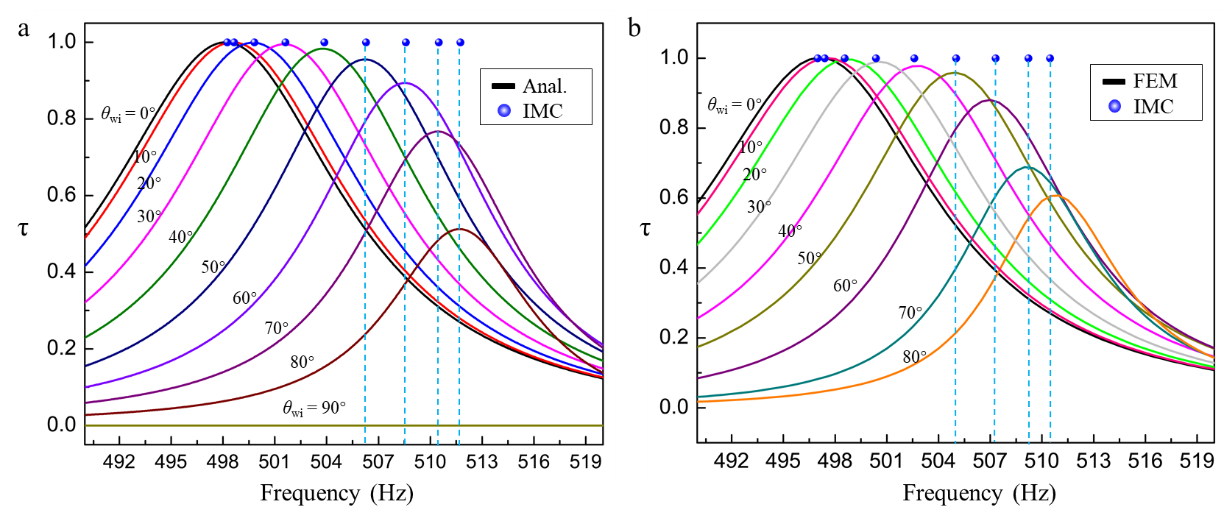


**Fig. S10. The analytical solution, IMC calculation and FEM calculation for water-to-air oblique incident agree well.** (**a**) The analytical solution for energy transmission coefficient at different frequencies in Note S12 agree well with the IMC calculation in Note S11. The parameters are *h* = 7.91 mm, *d* = 1.84 mm, and the maximum transmission frequency *f*_max_ at *θ*_wi_ = 0° is 498 Hz. According to the IMC calculations, the maximum transmission frequency *f*_smax_ at other incident angles can be obtained as , which agrees well with the analytical solution (the curves in **a**). (**b**) The FEM calculation (curves) agrees well with the IMC calculation (the dots at the frequency of the maximum transmission). The parameters are *h* = 7.91 mm, *d* = 1.84 mm, and the maximum transmission frequency for FEM at *θ*_wi_ = 0° is 497 Hz. The maximum transmission frequency at other incident angles can also be calculated with the equation above. The energy transmission coefficient does not equal 1 for the oblique incidence because the IMC for the normal incidence is not suitable for the oblique incidence. In the FEM calculations, the viscous and thermal losses are not considered.


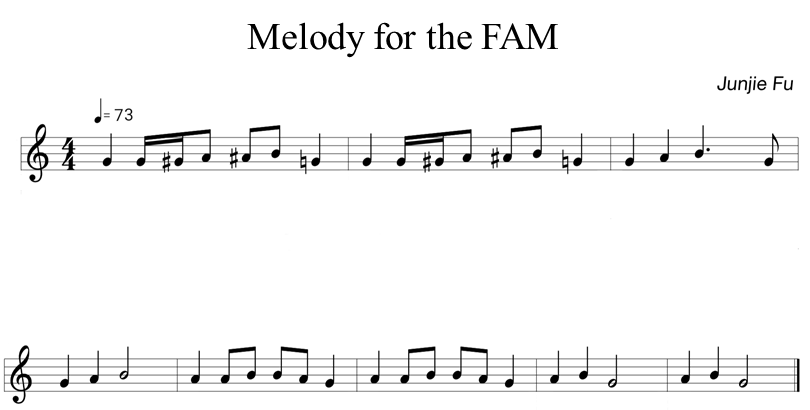


**Fig. S11. The melody of the music signal for the FAM.** The melody was made by playing the piano. The fundamental frequencies are at the range of 350 Hz - 500 Hz. The overtone frequency are shown in Fig. 6d

**Captions for Movie S1-10:**

**Movie. S1. The preparation of the fluid-type acoustic metasurface.** The solid structure was printed with the 3D printing technology. The material is nylon with a density of about 1020 Kg/m^3^ and the contact angle on the surface is about 115°. When being immersed in water, the hydrophobic surface enables it to trap bubbles in the hollow cells, and the density slightly larger than water makes it possible to be automatically immersed at a certain depth by controlling the gravity and buoyancy force, as shown in the video.

**Movie. S2. The FEM simulation of the formation of bubbles in Movie S1.** The two-dimensional simulation was used for simplifications. In the video, the curvature of the bottom air-water interface increases as immersion depth increases. Once the bubbles are formed, the curvature will decrease. This is because the process before the bubble formation is an isobaric process. The pressure in the hollow cell is always equal to atmospheric pressure. The structure requires to increase the curvature to increase the Laplace pressure to resist the increasing static pressure. After the formation of bubbles, the bubbles can increase the pressure by shrinking to overcome the liquid static pressure, therefore as the depth increase, the curvature doesn’t increase anymore.

**Movie. S3. The demonstration of the stability of the trapped bubbles with the shearing motion.** The bubble can be very stable after formation in the hollow cells. As shown in the video, the relative shearing motion between the bubbles and water have little influence on the trapped bubbles.

**Movie. S4. The demonstration of the stability of the trapped bubbles with the vertical motion.** As shown in the video, the relative vertical motion at a small speed doesn’t affect the stability of trapped bubbles. The theoretical calculation suggests that trapped bubbles can sustain a maximum speed of 0.67 m/s for the vertical motion between the bubbles and water.

**Movie. S5. The demonstration of the stability of the trapped bubbles with disturbance from water wave.** As shown in the video, the small water wave has little influence on the stability of the bubbles because there is little additional pressure acting on the bubbles.

**Movie. S6. The vertical immersion method could be used for the large *d*.** For the case that *d* is large and the parameters doesn’t satisfy the conditions for the horizontal immersion method, the vertical immersion method could be used if the parameters satisfy its conditions. In the video, the two structure with the same parameters doesn’t satisfy the conditions for the horizontal immersion method but satisfying that of the vertical immersion method. Therefore, the vertical immersion method is successful to trap bubbles.

**Movie. S7. The performance of the acoustic metasurface with the scanning frequency.**

The *f*_max_ in the experiment still agrees with the FEM when a small water sink is used. The parameters in the video are about *h* = 8.8 mm, *d* = 5.1 mm*, a* =3 mm, *w* = 1.2 mm. The two windows in the video are respectively the experimental operating platform and the measurement real-time signal. The frequency sweep signal increases linearly from 200 Hz to 700 Hz. It suggests that the experimental signal has a highest transmission near the peak frequency of about 452Hz.

**Movie. S8. The comparison between the case with and without the fluid-type acoustic metasurface (Movie with sound).**

the qualitatively transmission comparsion with and without the FAM is also presented. As shown in the video, the sound is much larger for the case that the solid structure is immersed in the water than the case of taking the bubbles out of the water. The bubbles can be still trapped into the solid structure when they are taken out of water, therefore the processes can be repeated again and again.

**Movie. S9. The FAM can work at different frequencies by changing the immersion depth.**

The noise source in a small sink was used to qualitatively present the performance of the FAM.

Due to the energy transmission coefficient is not sensitive to the value of *h*/*d*, the FAM can work at a large range frequency although the impedance matching condition isn’t be satisfied. The video shows that the transmission enhance effect exists at a wide frequency. Only by manually changing the immersion depth, the maximum transmission frequency can be changed.

**Movie. S10. The FAM can enhance the transmission of music signal across the water-air interface (Movie with sound).** The music signal was specially designed, with the fundamental frequencies from 350 Hz to 500 Hz, which is near the operation frequencies (*f*_max_ = 452 Hz) of the FAM. The movie shows, with the FAM, the music signal could be emitted from water to air. And without the FAM, the music signal was weakened. Then putting the air bubbles into the water again to form the FAM, the sound was enhanced again.

**Reference**

1. E. Bok, J. J. Park, H. Choi, C. K. Han, O. B. Wright, S. H. Lee, Metasurface for water-to-air sound transmission. *Phys. Rev. Lett.* **120**, 044302 (2018).
2. G. Ma, M. Yang, S. Xiao, Z. Yang, P. Sheng, Acoustic metasurface with hybrid resonances. *Nat. Mater.* **13**, 873-878 (2014).
3. Rienstra, S. W. & Hirschberg, A. An Introduction to Acoustics. *Eindhoven University of Technology* **18**, 19 (2004).
4. Rao, S. S. *Vibration of Continuous Systems*. Vol. 464 (Wiley Online Library, 2007).
5. Müller, P. C. & Schiehlen, W. *Linear Vibrations: A Theoretical Treatment of Multi-Degree-of-Freedom Vibrating Sems*. Vol. 7 (Springer Science & Business Media, 2012).
6. Hensel, R. *et al.* Wetting resistance at its topographical limit: the benefit of mushroom and serif T structures. *Langmuir* **29**, 1100-1112 (2013).
7. Whyman, G. & Bormashenko, E. How to make the Cassie wetting state stable? *Langmuir* **27**, 8171-8176 (2011).
8. De Gennes, P.-G., Brochard-Wyart, F. & Quéré, D. *Capillarity and Wetting Phenomena: Drops, Bubbles, Pearls, Waves*. (Springer Science & Business Media, 2013).
9. Huang, Z. *et al.* Bioinspired patterned bubbles for broad and low-frequency acoustic blocking. *ACS Appl. Mater. Interfaces* **12**, 1757-1764 (2019).
10. Young, D. F., Munson, B. R., Okiishi, T. H. & Huebsch, W. W. *A Brief Introduction to Fluid Mechanics*. (John Wiley & Sons, 2010).
11. Stinson, M. R. The propagation of plane sound waves in narrow and wide circular tubes, and generalization to uniform tubes of arbitrary cross‐sectional shape. *J. Acoust. Soc. Am.* **89**, 550-558 (1991).
12. Szabo, T. L. Lumped‐Element Transmission‐Line Analog of Sound in a Viscous Medium. *J. Acoust. Soc. Am.* **45**, 124-130 (1969).
13. Yu, N. *et al.* Light propagation with phase discontinuities: generalized laws of reflection and refraction. *Science* **334**, 333-337 (2011).
14. Assouar, B. *et al.* Acoustic metasurfaces. *Nat. Rev. Mater.* **3**, 460-472 (2018).
15. Kinsler, L. E., Frey, A. R., Coppens, A. B. & Sanders, J. V. *Fundamentals of Acoustics*. (1999).
16. Maznev, A., Every, A. & Wright, O. Reciprocity in reflection and transmission: What is a ‘phonon diode’? *Wave Motion* **50**, 776-784 (2013).
17. Gerjuoy, E. Refraction of waves from a point source into a medium of higher velocity. *Phys. Rev.* **73**, 1442 (1948).
